# How to Boost Positive Interpretations? A Meta-Analysis of the Effectiveness of Cognitive Bias Modification for Interpretation

**DOI:** 10.1371/journal.pone.0100925

**Published:** 2014-06-26

**Authors:** Claudia Menne-Lothmann, Wolfgang Viechtbauer, Petra Höhn, Zuzana Kasanova, Simone P. Haller, Marjan Drukker, Jim van Os, Marieke Wichers, Jennifer Y. F. Lau

**Affiliations:** 1 Department of Psychiatry and Psychology, South Limburg Mental Health Research and Teaching Network, EURON, Maastricht University, Maastricht, The Netherlands; 2 Department of Experimental Psychology, Oxford University, Oxford, United Kingdom; 3 Visiting Professor of Psychiatric Epidemiology King’s College London, King’s Health Partners Department of Psychosis studies Institute of Psychiatry, London, United Kingdom; 4 Psychology Department, Institute of Psychiatry, Kings College London, London, United Kingdom; University of Vienna, Austria

## Abstract

The current meta-analysis explores the strength of effects of cognitive bias modification training for interpretation bias (CBM-I) on positive (i.e., adaptive) interpretations and mood as well as the training and sample characteristics influencing these effects. Data-bases were searched with the key words “interpret* bias AND training” and “interpret* bias AND modif*”. Reference lists of identified articles were checked and authors of identified articles were contacted for further relevant articles and unpublished data. Studies were reviewed for inclusion with eligibility criteria being that the study (a) aimed to target interpretation biases through any kind of training, (b) assessed mood and/or interpretation bias as outcome measures, (c) allocated individuals to training conditions at random, and (d) recruited adult samples. A meta-analytic multilevel mixed-effects model was employed to assess standardized mean changes in interpretation bias, negative mood, and emotional reactivity. In addition, several training and sample characteristics were explored for their potential to enhance benign training effectiveness. On average, benign CBM-I resulted in an increase in positive interpretation bias (*p*<.01) and a decrease in negative mood state (*p*<.001), but did not affect emotional reactivity. These effects were not consistently different from control conditions with no or neutral training. However, within benign training conditions imagery instructions and more training sessions were related to larger cognitive and mood effects, whereas feedback about training performance and inclusion of non-benign training items (instead of including benign items only) boosted cognitive effects only. Finally, training was more effective in women (cognitive *and* mood effects) and presumably samples with symptomatic emotional dysregulation (cognitive effects). Although the effects of emotional dysregulation and number of training sessions could not well be distinguished, there is an indication that when used with imagery instructions and more training sessions, benign CBM-I can be employed as a useful complementary treatment to usual psychotherapies.

## Introduction

Clinically relevant anxiety and depression are common, affecting up to 30% of individuals during their lifetime [1], [2]. These conditions are distressing, disruptive, and costly [1], prompting calls for more research into their treatment and prevention. Cognitive bias modification (CBM) training may be able to partially address this need. CBM training aims to modify information-processing biases linked to anxiety and depression through computerized, repeated practice that reinforces more adaptive styles of processing. An added benefit of CBM is that it can be implemented with relative ease. While recent individual studies appear promising, questions remain on the degree to which CBM can actually ‘correct’ biases and reduce symptomatology.

CBM for interpretation biases (CBM-I) focuses on modifying interpretation biases and draws on extant data from cognitive psychology showing that anxiety and depression are characterized and maintained by negative interpretation biases. This is the tendency to draw negative interpretations from ambiguous stimuli [3], [4]. CBM-I capitalizes on these findings by aiming to ‘train’ more adaptive interpretational styles. There are three main training methods: the homograph paradigm [5], the word-sentence association task (WSAT) paradigm [6], and, perhaps most widely-used, the ambiguous situations (AS) paradigm [7]. In each paradigm, ambiguous stimuli are presented to participants across multiple trials – with trials ending with a response from the participant that resolves the stimulus in a benign direction. For example, stimuli can be ambiguous homographs, which are words that have both a negative and a benign meaning (e.g., ’patient’, which can be interpreted as a doctor’s patient implying something negative like illness, but can also be interpreted as the human capacity of endurance implying a positive character trait), or ambiguous sentences and situations, which can be interpreted in either a negative or benign manner (e.g., “*Having finished painting the lounge, you invite friends around to dinner. As they walk into the room, you can see that they are surprised. Their reaction is one of…”* can be interpreted to mean that the friends are pleased or displeased). Stimulus presentations are then followed by a “probe” to which the participants must respond. Usually these “probes” are word fragments, which must be completed by the participant by indicating the first missing letter (e.g., in case of the ambiguous situation described above, this could be pl–sure for pleasure or h–rr–r for horror). By correctly completing these words, the ambiguity of the stimulus is resolved in a benign direction for the benign CBM-I condition. During comparison conditions, the stimuli are resolved negatively or neutrally. Usually, the word fragments are presented in such a way that there is only one possible solution. To further encourage a valenced interpretation of the ambiguous stimulus, trainings have made use of adding a comprehension question (e.g., in case of the ambiguous situation described above, this could be “Did your friends like your lounge?” with a correct ‘Yes’-response for the benign and ‘No’-response for the negative training condition) and feedback about response accuracy [7].

Training effectiveness is typically assessed with either change in measures of interpretation biases and mood or after training only. Additionally, assessing subsequent mood-reactivity to emotional challenges aims to address whether CBM-I is able to impact on exuberant increase in negative mood in response to stressful stimuli. Several emotional challenges have been employed to investigate whether CBM-I can impact on responses to ‘stress’ stimuli. Stressors have included videos showing accidents, e.g., see [8], unsolvable anagram tasks, e.g., see [9], or negative mood inductions, e.g., see [10]. While earlier CBM-I studies used training methods to investigate whether generating benign and negative interpretations would, in unselected/healthy samples, alter mood and/or mood reactivity, more recent studies have assessed the suitability of these training methods to *improve* anxious and depressive moods in subclinical and clinical samples.

Other than individual studies, there is initial meta-analytic evidence for the effectiveness of benign CBM-I. Hallion and Ruscio [11] investigated the effects of CBM-I in combination with another training package, CBM for attention (CBM-A). They reported significant and large post-training differences in interpretation biases and mood states of benign CBM-I as compared to control conditions [11]. Although these findings are very encouraging, some further elaboration is required to address the potential clinical effectiveness of benign CBM-I for several reasons. First, based on the results above, the possibility remains that a pre-existing tendency to select positive over negative interpretations (i.e., an optimistic bias) can account for the large post-training differences between the benign and control conditions. Although participants were randomly assigned to training conditions, significant post-training differences between training conditions could also have arisen solely from *trained* differences in the opposite direction among the comparison groups. Comparison groups have included ‘no training’ to control for natural fluctuations for that sample on outcome measures; ‘neutral training’ (where there is roughly an equal number of benign and negative resolutions across training items) to control for possible ‘placebo’ effects of CBM-I training; and ‘negative training’ (where consistently negative resolutions of ambiguity are presented) to explore whether post-training differences between benign and negative training are driven by negative changes in the negative training, positive changes in the benign training, or both. If post-training differences between groups [11] were mainly driven by negative *changes within* the comparison groups, the clinical benefit of benign CBM-I would be questionable.

Second, although the existing meta-analysis [11] suggests significant post-training differences between groups on anxiety and depression symptoms, the effects were rather small after an emotional challenge [11]. Anxiety and depression symptoms were conceptualized by merging mood-measures and more elaborate symptom-measures such as the Hamilton Anxiety Rating Scale or the Beck Depression inventory. However, several symptom scales were developed for use *within* clinical samples only and may vary minimally in undiagnosed participants [12], [13] – with whom the majority of CBM-I studies have been conducted [11]. Furthermore, findings on training effects on symptoms have been mixed in individual studies, e.g. see [8], [14], and finally, changes in symptoms such as rumination immediately after CBM-I – even in clinical samples – may be considered unlikely. In contrast, continuous variation in mood states not only may be used to characterize the common symptom ‘low mood’ of anxiety and depression but also can be measured reliably in general population samples [15], [16], particularly in response to emotional challenges [17]. Clearly addressing the effects on CBM-I on mood-states only would help drawing a clearer picture about its potential clinical effectiveness.

Besides expanding Hallion & Ruscio’s pioneering meta-analysis by addressing both the change within the benign condition and the effects on mood only, we most of all need to know how to optimize effects and pin down the factors that make it effective [11]. It is currently unclear whether one of the various CBM-I paradigms used (i.e., homograph, AS, and WSAT) is more effective than others. It has also been suggested by individual studies that training is more effective when participants have to (a) *generate* words and meaning of ambiguity themselves instead of simply being exposed to them [7], and (b) *imagine* the situations happening to themselves instead of processing stimuli more passively [10], [18]. Furthermore, it has been proposed that the administration of feedback about response accuracy will reinforce participants “for making valenced interpretations” (p.606) [7]. However, to the best of the authors’ knowledge, this has not yet been systematically tested.

Additional training characteristics, such as the mode of stimuli presentation (visual or auditory), and the *ratio* of training items in the benign training direction to the total number of presented items (i.e., does the fraction of benign items matter?) also frequently vary between CBM-I studies but have not yet been systematically investigated. Recent studies have also specifically assessed the potential clinical use of CBM-I across several training sessions [6], [19], [20], suggesting that multiple sessions will increase effectiveness. Finally, it is commonly accepted that people with emotional symptoms suffer from negative interpretation biases, e.g., see [3], [4] and women present more often with emotional problems than men, e.g. see [21]. It is therefore of clinical relevance to investigate whether benign CBM-I is particularly effective in symptomatic and female samples.

In the current meta-analysis, we first explore benign CBM-I as a possible clinical tool by examining the within group *change* in interpretational style and mood state and second, we assess factors that increase the effectiveness of benign CBM-I. Because we are primarily interested in the potential benefits of benign CBM-I, we conceptualized changes in interpretation bias as increases in positive interpretations (i.e., defined as an adaptive interpretation bias encompassing both benign and explicit positive interpretation styles) as opposed to decreases in negative interpretations. Indeed, previous research has shown that healthy individuals more likely draw positive than negative interpretations of ambiguous situations, whereas individuals with a current anxiety disorder are more likely to draw almost as many positive as negative interpretations [22]. Therefore, the difference between positive and negative interpretation bias after training has been of great interest to researchers and these indices were reported in the majority of studies. We therefore also assessed the post-training endorsement of positive versus negative interpretations.

Next, we assessed changes in negative mood from pre- to post-training as well as in response to an emotional challenge. In a second step, we compared these changes/differences in the benign CBM-I group with various control conditions and explored the degree to which the *change* in interpretation bias was associated with the *change* in mood in response to benign CBM-I training. To the extent that benign CBM-I was effective at changing interpretational style and mood within the benign condition, we investigated whether this effect varied across various training characteristics but also across sample characteristics, such as age, sex, and the inclusion of high symptomatic individuals.

## Methods

### Protocol

The protocol for reviewing the articles was developed by the first author based on the suggested strategy by Lipsey and Wilson [22] and adhered to the PRISMA guidelines (Table S1). Overall, the protocol consisted of a detailed description of the criteria to be employed for the search strategy, deciding on study eligibility, coding of the necessary variables, and procedures for resolving disagreements in coding (please see below ‘eligibility criteria’, ‘information sources and search’, ‘study selection’, ‘data collection process’, and ‘data items’ for the detailed description).

### Eligibility criteria

Eligibility was assessed based on five criteria. First, the study aimed to target interpretation biases through any kind of training. Second, for studies with more than one training group, allocation to training groups was done at random to prevent confounding of the data by any third variables such as personal preference. Third, interpretation biases and/or mood state had to be assessed as the outcome measure to allow the assessment of training effectiveness. Fourth, the sample consisted of healthy adults, adults with subclinical/high-trait symptoms, or adults with clinical diagnoses of any anxiety or major depressive disorder. We did not include studies of child and adolescent samples as the relationship between cognition and mood may vary across development [23]. Fifth, studies had to be published in English, German, or Dutch. All identified publications including articles, conference abstracts, and dissertation abstracts were considered eligible.

### Information sources and search

In November and December 2010, databases (i.e., EMBASE, Medline, PsychArticles, Psychology & Behavioral Science Collection, PsycINFO, Science Direct, and Web of Science) were searched simultaneously with the key words “interpret* bias AND training” and “interpret* bias AND modif*” for publications in this area between 1992 and 2010. To the authors’ knowledge, CBM-I was first introduced in 2000; additional searches for the years 1992 through 1999 were conducted to ensure that no earlier and possibly less popular accounts were missed. All reference lists of identified articles were cross-checked for further relevant articles. All authors of identified articles were also contacted with a request to send any additional relevant literature and/or unpublished data that might be appropriate for inclusion into the meta-analysis. Finally, follow-up literature searches were conducted in October 2011 and June 2013 for studies published since our first and second search.

### Study selection

All hits were screened in a standardized fashion adhering to the PRISMA guidelines (Preferred Reporting Items for Systematic Reviews and Meta-analyses; [24]) involving: screening the titles, abstracts, and finally the whole article for eligibility criteria. The whole screening procedure was conducted independently by two researchers (CML and PH for the first two searches, and CML and SH for the last search). If researchers did not agree on inclusion, the article was nevertheless included in the next screening stage to minimize chances of premature exclusion. All articles fulfilling eligibility criteria were included in the meta-analysis if sufficient data were available (either provided in the text or by researcher on request).

### Data collection process

All included articles were independently coded by at least two researchers (CML, PH, ZK, and SH) using a standardized coding protocol and appropriate forms (available upon request from the first author; also see ‘protocol’). The summary statistics necessary to compute the effect sizes (see below) were retrieved from the articles by at least two independent coders (CML, PH, ZK, and SH), compared, and if necessary adapted. All missing data were requested from authors via e-mail. As most primary articles report between-group differences at post-training rather than within-group comparisons, almost all authors were contacted. All but five out of 35 of those contacted responded positively (response rate = 86%).

### Data items

Articles were first coded on the basis of inclusion of the different training conditions used. We distinguished between four conditions: (i) Benign training, where the majority of ambiguous stimuli were resolved in a positive or non-negative direction; (ii) Negative training, where the majority of ambiguous stimuli were resolved in a negative, threatening, or harmful direction; (iii) Neutral training, where ambiguous stimuli were resolved in an overall neutral direction (either by presenting the same amount of stimuli in benign and negative directions, or by resolving stimuli in a neutral direction); and (iv) no training, in which participants were not exposed to any form of interpretation bias training but were simply tested and re-tested on selected outcome measures. Outcome measures were categorized into one of three categories, namely cognition (i.e., any kind of interpretation bias assessment), mood, or other. For each available outcome measure, (a) the sample size (N or df), (b) the mean before and after training or the mean difference between before and after training, (c) the standard deviation, variance, or standard error for before and after training or for the difference between before and after training, and (d) the correlation or the dependent sample t-value between before and after training were recorded.

Articles were also coded with regard to potential moderators, consisting of sample and training characteristics. Sample characteristics included age (mean age of whole sample), sex (percentage of men in the whole sample), and presence of high levels of anxiety and depressive symptoms (including clinical diagnosis) in participants. Training characteristics included the type of training paradigm used (AS, homograph, WSAT, or other), format of training (generation of the meaning of words and situations versus simple exposure), pre-training instructions (presence or absence of the use of mental imagery), modality of training (visual or auditory), use of feedback (presence or absence of feedback about participant’s response (correct/incorrect)), the training ratio (ratio between the number of stimuli in the training direction to the total number of stimuli), and frequency (the total number of training sessions).

### Risk of bias in individual studies

Risk of bias in individual studies was attempted to be kept at a minimum by making randomization to training condition an inclusion criteria for those reports including more than one training condition. For those studies that administered training across several sessions, attrition (percentage of drop-out) within each training condition was coded as a proxy for risk of selected attrition in the benign condition.

### Summary measures

Individual effect size estimates were computed for each study and for each training condition separately across the following four (within-group) contrasts: (i) post-training endorsements of positive versus negative interpretations; (ii) pre-training versus post-training endorsements of positive interpretations; (iii) pre-training versus post-training negative mood ratings; and (iv) pre-emotional challenge versus post-emotional challenge negative mood ratings. Please note that ‘positive’ interpretations were defined as including both benign and ‘non-threat’ as well as explicit positive interpretations. As the effect size measure for the meta-analysis, we used the standardized mean difference for the difference between positive versus negative interpretation endorsements post-training (contrast i) and the standardized mean change for the pre- to post-training/emotional challenge contrasts (contrasts ii through iv). Standardization was based on the differences and change scores, respectively [25].

In particular, the standardized mean change for each condition was computed with *d* = Mean_diff_/SD_diff_, where Mean_diff_ denotes the mean of the change scores, SD_diff_ = *√(*SD^2^_pre_+SD^2^_post_ - 2*r*SD_pre_SD_post_), and *r* denotes the correlation between the pre- and post-training/emotional challenge assessments (for the contrast of the endorsements of the positive versus negative interpretations, Mean_diff_ denotes the mean of the endorsement differences, SD_diff_ = √(SD^2^_positive_bias_+SD^2^_negative_bias_ - 2*r*SD_positive_bias_SD_negative_bias_), and *r* denotes the correlation between the endorsements of the positive and negative interpretations). If SD_diff_ was not reported, the paired-samples t-test value was employed to calculate the effect size with *d* = *t/√n*. The sampling variance of the d-values was calculated with *v* = 1/*n*+*d^2^*/2*n,* where *n* denotes the group size.

Sometimes a study would provide sufficient information to compute multiple *d*-values for the same sample for a particular contrast (e.g., when more than one mood scale was used to assess mood state pre- and post-training). To avoid the problem of non-independent effect size estimates in these samples, we selected only one measure based on an *a priori* established preference list [26]. In general, measures employed more often and assessing the outcome construct more directly were preferred over other measures (see Table S2). Moreover, if the same group of subjects underwent more than one training (whether it be a different training condition or a variation of the same training condition) we only computed *d* for the first training condition the group was exposed to. On the other hand, if different groups of subjects underwent slight variations of the same training condition within the same study (e.g., when one group was exposed to benign training with *visual* stimuli presentation and another group was exposed to benign training with *auditory* stimuli presentation), then multiple *d*-values for that training type (e.g., benign training) could be extracted (while still preserving the statistical independence of the *d*-values).

Correlations between the pre- and post-training/challenge measurements (or post-training positive and negative interpretations) were calculated for all studies that reported the necessary values. Of note, *r* can be inferred when only SD_diff_, SD_pre_, and SD_post_ are known. For studies where *r* was unknown and SD_diff_ had to be computed in order to obtain *d*, the mean correlation was employed to impute *r* (this was done separately for each of the four training conditions per contrast). Therefore, instead of leaving out these studies, this approach allowed us to include more samples in the meta-analysis, namely another 17 samples for post-training positive versus negative endorsements, 12 samples for change in negative mood, and two samples for the increase in negative mood in response to an emotional challenge.

To summarize, for each of the four key contrasts (i.e., interpretation bias as assessed with post-training positive versus negative interpretation endorsements; interpretation bias as assessed with change in positive bias from pre- to post-training; immediate change in mood state pre- to post-training; and change in mood state in response to an emotional challenge) a set of effect size estimates across studies was obtained, describing the degree of the difference or the amount of change for each training condition. Depending on the number of independent training conditions employed by a primary study, one, two, or more *d*-values could be extracted for a particular contrast from each study.

### Synthesis of results

#### Changes within groups and comparison between groups

Due to the multilevel structure of the data (with multiple effect size estimates nested within the studies), we used a meta-analytic multilevel mixed-effects model analogous to the one described by Salanti et al. [27] for the analyses. In particular, random effects were added at the study level (to account for the fact that the size of the effects may generally be larger or smaller across all conditions examined within a study) and at the effect size level (to account for heterogeneity in the size of the treatment effects). Dummy variables for the four different training conditions were added to the model, so that we could estimate the (average) standardized mean change/difference for each training condition. Furthermore, we could then compare these changes/differences *within* each training condition *between* the four training conditions, yielding six pairwise comparisons (benign-neutral, benign-no training, benign-negative, neutral-no training, neutral-negative, no-training-negative) to control for natural fluctuations for that sample on outcome measures (benign-no training comparison), for possible ‘placebo’ effects of CBM-I training (benign-neutral training comparison), and to explore whether post-training differences between benign and negative training are driven by negative changes in the negative training, by actual positive changes in the benign training, or by both (benign-negative training comparison).

An omnibus Wald-type test was used to test for any differences between the four training conditions. Similarly, the average standardized mean change for each training condition and the pairwise contrasts were tested for significance at α = .05 (two-sided). We also report 95% confidence intervals for the estimated averages and pairwise contrasts. Finally, likelihood ratio tests were conducted to test whether the variance in the random effects at the study and the effect size level was significantly greater than zero.

Significant heterogeneity at the study level indicates that, due to nonspecific study characteristics, effect sizes for all training conditions can be larger or smaller in one study than another. Significant heterogeneity at effect size level indicates that the effect of a particular training condition is not constant across studies. Therefore, if significant heterogeneity at the study or effect size level for a particular outcome is found, then the pooled effect sizes based on the meta-analytic models need to be interpreted as depicting the *average* size of the effects.

#### Factors influencing changes within benign CBM-I

Given our interest in factors that enhance the effects of benign training, secondary meta-regression analyses were conducted to examine potential moderating effects of the various sample and training characteristics. To reduce the number of tests, these analyses were only conducted for the benign training condition, given our *a priori* interest in the effectiveness of this particular training type. Moreover, only contrasts that yielded a significant effect in primary analyses were explored further in these secondary analyses.

The first moderator was training paradigm, in which we distinguished between the ambiguous situations (AS) task versus all other paradigms combined. We did this for two reasons: (i) the AS task was most widely employed, whereas few studies employed the WSAT and homograph task for particular outcomes, and (ii) the AS task has the highest ecological validity, as it describes common everyday life ambiguous situations and might therefore more directly relate to people’s real lives than words or sentences (that are employed in the other training paradigms).

Next, we explored training characteristics as moderators, including use of *imaging* instructions (present/absent), instructions to *generate* meaning (present/absent), inclusion of *feedback* (present/absent), and mode of *presentation* (auditory/visual). Sample characteristics that were also examined included: participant *status* (healthy/symptomatic), coded dichotomously, and *age, sex* (proportion men), training *frequency* (nr. of training sessions), and *ratio* of training items in the intended training direction (benign or negative) to the total number of presented items, included as continuous variables in the meta-regression models.

For dichotomous variables, we compared effect sizes for the presence and absence of the dichotomous variables (auditory as compared to visual, symptomatic as compared to healthy, for *presentation* and *status*, respectively), reflecting the difference in effect size for the two levels of the moderator. For continuous variables, we assessed whether greater levels of potential moderators enhanced or attenuated the effects of the benign training condition, reflected by the change in the size of the effect for a one-unit increase in the moderator.

Each of the moderators was added to the meta-regression separately (we were unable to enter the moderators simultaneously as the majority of data would be lost due to missing values (also see table 1), which would have drastically reduced the power to find any relationships). However, since training paradigm (AS, homograph, and WSAT) is a rather complex variable possibly differentially influencing the effect of other training characteristics, we controlled for type of paradigm (whenever training paradigm resulted in a significant moderating effect) when analyzing the more specific training characteristics. Therefore, we investigated whether any given training-characteristic moderated training effectiveness *above* and *beyond* the training paradigm employed. Additionally, we examined the size of the correlations for all other moderators to explore whether any of these were strongly associated. If two moderators were strongly correlated and both revealed a significant effect, it would be difficult to conclude which of the two moderators underlies this effect.

**Table 1:** Study descriptives.

General								Outcome measures			Training conditions					
Article	study	healthstatus^1^	trainingparadigm^2^	meanage	% male(sex)	Trainingitems/totalitems (ratio)^3^	No ofsessions(frequency)	interpretation bias	mood-state	emotionalchallenge	condition	N	imageryinstructions (y/n)	wordgeneration (y/n)^4^	mode ofpresentation^5^	Feedbackadministration (y/n)^6^
Amir et al(2010)	1	anxious	WSAT	19,50	47	1,00	1	IB questionnaire	STAI-S	NA	benign	29	n	n	visual	Y
											neutral	28	n	n	visual	Y
Amir & Taylor(2012)	1	anxious	WSAT	31,00	29	1,00	12	WSAT	STAI-T/LibowitzAnxiety Scale/SPAI	NA	benign	20	NA	n	visual	Y
											neutral	23	NA	n	visual	Y
Beard & Amir(2008)	1	anxious	WSAT	20,00	7	0,69	8	WSAT	SPAI, STAI-T	NA	benign	13	n	n	visual	Y
											neutral	14	n	n	visual	Y
Blackwell &Holmes (2010)	1	depressed	AS	37,70	29	1,00	7	VAS depressive bias, SST	PAS, NAS	NA	benign	7	Y	n	auditory	n
Bowler et al(2012)	1	anxious	AS	22,70	32	1	4	SST	STAI-T/FNES	NA	benign	21	Y	Y	visual	Y
											no training	21	NA	NA	NA	NA
Clerkin et al(2011)	1	anxious	AS	18,76	35	0,92	1	similarity ratings	NAS	car accidence sentenceabout best friend	benign	50	Y	Y	visual	NA
											neutral	49	Y	Y	visual	NA
Grey & Mathews(2000)	1	healthy	homograph	NA	45	0,33	1	RT word fragment	NA	NA	negative	20	n	Y	visual	Y
											benign	20	n	Y	visual	Y
	2	healthy	homograph	NA	NA	0,40	1	lexical decision task	NA	NA	negative	20	n	Y	visual	Y
											benign	17	n	Y	visual	Y
	3	healthy	homograph	NA	NA	0,40	1	lexical decision task	NA	NA	negative	20	n	n	visual	Y
											benign	20	n	n	visual	Y
	4	healthy	homograph	NA	NA	0,00	1	lexical decision task	NA	NA	neutral	20	n	n	visual	Y
Grey et al(2009)	1	healthy	homograph	NA	45	0,40	1	lexical decision task	NA	NA	benign	18	n	n	visual	Y
											negative	19	n	n	visual	Y
Hayes et al(2010)	1	anxious	homograph& AS	42,00	23	0,80	1	NA	VAS anxiety/depression/happy	worry intrusion	benign	20	n	both	both	Y
											neutral	20	n	both	both	Y
Hertel et al(2003)	1	healthy	homograph	NA	33	0,40	1	form image	NA	NA	no training	18	n	n	visual	Y
											negative	18	n	n	visual	Y
											benign	17	n	n	visual	Y
	2	healthy	homograph	NA	36	0,40	1	form image	NA	NA	benign	22	n	n	visual	Y
											negative	22	n	n	visual	Y
Hertel et al(2011)	1	healthy	AS	NA	50	0,78	1	NA	VAS happy/distress/tension/pessimism	NA	negative	16	y	Y	visual	NA
											neutral	16	Y	Y	visual	NA
											benign	16	Y	Y	visual	NA
	2	anxious	AS	NA	50	0,78	1	NA	VAS happy/distress/tension/pessimism	video stressor	benign	20	Y	Y	visual	NA
											neutral	20	Y	Y	visual	NA
Hirsch et al(2007)	1	healthy	AS	NA	17	0,90	1	NA	STAI-S, NAS	NA	negative	12	n	N	visual	Y
											benign	12	n	N	visual	Y
Hirsch et al(2009)	1	anxious	homograph& AS	35,65	20	0,86	1	NA	VAS anxiety/depression/happy	worry intrusion	neutral	20	n	both	both	Y
											benign	20	n	both	both	Y
Holmes et al(2006)	1	healthy	AS	38,85	35	1,00	1	emotionality ratings	STAI-S, PAS	NA	benign	13	n	n	auditory	n
											benign	13	Y	n	auditory	n
Holmes et al(2009)	1	healthy	AS	30,98	45	1,00	1	emotionality ratings	STAI-S, STAI-T, PAS	negative moodinduction	benign	20	Y	n	auditory	n
											benign	20	n	n	auditory	n
	2	healthy	AS	24,95	33	1,00	1	emotionality ratings	STAI-S, PAS	NA	benign	20	Y	n	auditory	n
											benign	20	n	n	auditory	n
	3	healthy	AS	24,95	33	1,00	1	emotionality ratings	STAI-S, PAS	NA	benign	20	n	n	auditory	n
Hoppitt et al(2010a)	1	healthy	AS	35,61	39	1,00	1	emotionality ratings	STAI-S	NA	negative	14	Y	Y	visual	Y
											negative	14	Y	n	visual	Y
Hoppitt et al(2010b)	1	healthy	homograph	42,21	39	1,00	1	RT word fragment	STAI-S	video stressor	negative	22	n	Y	visual	n
											benign	25	n	Y	visual	n
Lang et al(2009)	1	healthy	AS	27,67	48	0,90	1	similarity ratings	PAS, NAS	video stressor,picture from video	benign	24	Y	Y	visual	Y
											negative	24	Y	Y	visual	Y
Lang et al(2012)	1	depressed	AS & other	28,45	23	1,00	6	SST, RIQ	STAI-T	NA	neutral	13	Y	both	both	n
											benign	13	Y	both	both	
Lange et al(2010)	1	anxious	AS	20,71	12	0,69	1	similarity ratings	STAI-S, LibowitzAnxiety Scale	NA	benign	34	Y	Y	visual	Y
											negative	34	Y	Y	visual	Y
	2	anxious	AS	20,98	18	0,69	1	similarity ratings	STAI-S, LibowitzAnxiety Scale	NA	benign	18	Y	Y	visual	Y
											negative	16	Y	Y	visual	Y
MacDonaldet al. (2013)	1	anxious	WSAT	32,79	4	1	1	BBSIQ	Anxiety sensitivityindex	NA	benign	17	n	NA	visual	Y
											neutral	17	n	NA	visual	Y
Mackintoshet al (2006)	1	healthy	AS	NA	29	0,90	1	similarity ratings	STAI-S	NA	negative	24	Y	Y	visual	Y
											benign	27	Y	Y	visual	Y
	2	healthy	AS	NA	28	0,90	1	similarity ratings	STAI-S	accident video	benign	10	Y	n	visual	Y
											benign	10	Y	n	auditory	Y
											negative	10	Y	n	visual	Y
											negative	10	Y	n	auditory	Y
Mathews &Mackintosh(2000)	1	healthy	AS	NA	NA	0,69	1	similarity ratings	STAI-S	NA	negative	10	Y	Y	visual	Y
											benign	10	Y	Y	visual	Y
	2	healthy	AS	NA	NA	0,69	1	similarity ratings	STAI-S	NA	negative	10	Y	n	visual	Y
											benign	10	Y	n	visual	Y
	3	healthy	AS	NA	NA	NA	0	similarity ratings	NA	NA	no training	12	NA	NA	NA	Y
	4	healthy	AS	NA	NA	0,88	1	similarity ratings	STAI-S	NA	negative	13	Y	Y	visual	Y
											benign	13	Y	Y	visual	Y
	4a	healthy	AS	NA	NA	0,88	1	similarity ratings	STAI-S	NA	negative	13	Y	Y	visual	Y
											benign	13	Y	Y	visual	Y
	5	healthy	AS	NA	NA	0,60	1	similarity ratings	STAI-S	NA	negative	14	Y	Y	visual	Y
											negative	14	Y		visual	Y
											benign	14	Y	Y	visual	Y
											benign	14	Y	n	visual	Y
Mathews et al(2007)	1	anxious	homograph	40,65	31	0,80	4	reason for events, similarity ratings	STAI-S, STAI-T	NA	benign	19	Y	n	visual	Y
											no training	20	NA	NA	NA	NA
Murphy et al(2007)	1	anxious	AS	20,60	26	1,00	1	similarity ratings	STAI-S	NA	benign	22	Y	n	auditory	Y
											benign *	22	Y	n	auditory	Y
											neutral	22	Y	n	auditory	Y
Salemink et al(2007a)	1	healthy	AS	20,80	24	0,69	1	similarity ratings	STAI-S	unsolvable anagram	benign	60	Y	Y	visual	Y
											negative	58	Y	Y	visual	Y
Salemink et al(2007b)	1	healthy	AS	21,10	NA	0,69	1	similarity ratings, EAST,IB questionnaire	STAI-S, STAI-T	NA	benign	40	Y	Y	visual	Y
											negative	41	Y	Y	visual	Y
Salemink et al(2009)	1	anxious	AS	21,30	18	0,69	8	similarity ratings, IBquestionnaire	STAI-S, STAI-T, FNES	unsolvable anagram	benign	17	Y	Y	visual	Y
											neutral	17	Y	Y	visual	Y
Salemink et(2010a)	1	healthy	AS	21,10	9	0,69	1	NA	VAS positive/negative	mood induction	benign	21	Y	Y	visual	Y
											negative	20	Y	Y	visual	Y
Salemink et al(2010b)	1	healthy	AS	20,05	25,3	0,75	1	NA	STAI-S	NA	negative	38	Y	Y	visual	Y
											benign	37	Y	Y	visual	Y
Salemink et al(2010c)	1	healthy	AS	20,35	18	0,69	1	similarity ratings, IBquestionnaire	NA	NA	benign	21	Y	Y	visual	Y
											negative	20	Y	Y	visual	Y
											no training	51	NA	NA	NA	NA
	2	healthy	AS	20,70	12	0,69	1	similarity ratings	NA	NA	benign	34	Y	Y	visual	Y
											negative	34	Y	Y	visual	Y
Standage et al(2009)	1	healthy	AS	22,08	21	1,00	1	similarity ratings	VAS anxiety/depression	anxietyanticipation, speech	benign	12	Y	n	visual	Y
											benign	12	Y	n	auditory	Y
											negative	12	Y	n	visual	Y
											negative	12	Y	n	auditory	Y
Standage et al (2010)	1	healthy	other	17,60	17	1,00	1	SST	VAS anxiety/depression	NA	negative	14	n	n	visual	n
											benign	14	n	n	visual	n
Steel et al(2010)	1	anxious	AS	43,00	71	1,00	1	emotionality ratings	STAI-S	NA	benign	11	Y	n	auditory	n
											neutral	10	Y	n	auditory	n
Steinman et al(2010)	1	anxious	AS	18,93	31	0,69	1	NA	PANAS FS	anxiety-symptomsprovoking breathing task	no training	25	NA	NA	NA	NA
											benign	25	Y	Y	visual	Y
											neutral	25	Y	Y	visual	Y
Teachman et al(2008)	1	anxious	AS	18,60	26	0,69	1	similarity ratings	NAS	NA	benign	20	Y	Y	visual	Y
											neutral	20	Y	Y	visual	Y
											no training	20	NA	NA	NA	NA
Tran et al(2011a)	1	healthy	AS	NA	52	0,69	1	similarity ratings	NAS	NA	benign	29	Y	Y	visual	NA
											negative	29	Y	Y	visual	NA
Tran et al(2011b)	1	healthy	AS	NA	62	0,69	1	similarity ratings	PAS, NAS	emotional faces/incorrect feedback	benign	25	Y	Y	visual	NA
											negative	25	Y	Y	visual	NA
Turner et al(2011)	1	anxious	AS	24,75	88	1,00	1	NA	VAS mood	NA	benign	8	Y	Y	visual	NA
Williams et al(2013)	1	depressed	AS	44,80	7,5	1,00	7	SST	NA	NA	benign	26	Y	n	auditory	n
											no training	27	NA	NA	NA	NA
Wilson et al(2006)	1	healthy	homograph	18,15	50	1,00	1	RT word fragment	VAS anxiety/depression	video stressor	negative	24	n	Y	visual	Y
											benign	24	n	Y	visual	Y
Yiend et al(2005)	1	healthy	AS	32,50	40	0,90	1	similarity ratings	STAI-S, STAI-T	NA	benign	10	Y	Y	visual	Y
											negative	10	Y	Y	visual	Y
	2	healthy	AS	42,90	29	0,90	1	similarity ratings	STAI-S, STAI-T	NA	benign	12	Y	n	visual	Y
											negative	12	Y	n	visual	Y
	3	healthy	AS	39,60	37	0,90	1	similarity ratings	STAI-S, STAI-T	NA	benign	10	Y	Y	visual	Y
											negative	9	Y	Y	visual	Y

*Note*. y-YES, n-No; VAS = visual analogue scale; IB questionnaire = interpretation bias questionnaire, STAI-T/S = State Trait Anxiety Inventory – Trait/State; SST = Scrambled Sentence Test; PANAS = positive and negative affect scales; PAS = positive affect scale (from PANAS); NAS = negative affect scale (from PANAS); PANAS FS = fear subscale from PANAS; RT = reaction time; RIQ = Response to intrusions questionnaire, FNES = Fear of Negative Evaluation Questionnaire; EAST = extrinsic affective Simon task; NA = not available; * Murphy et al (2007) employed two independent benign groups that differed in that one group focused on emotional outcomes that were non-negative whereas the other group focused on pure positive outcomes. As this distinction was not made in the current meta-analysis both these training conditions were included under the ‘benign’ training condition.

1 health status = healthy, anxious, depressed (note: this considers symptoms and clinical diagnoses); ^2^AS = ambiguous situations paradigm, WSAT = word sentence association task, ‘other’ paradigms included picture word interpretation, sentence completion (Lang et al, 2011), positive or negative valenced statements (Standage, 2010); ^3^training items/total items (ratio) = number of items in training direction divided by total number of items in task; ^4^word generation = were participants required to actively generate emotional meaning?; ^5^mode of presentation = were stimuli presented visually or auditory?; ^6^feedback administration = was feedback about response accuracy administered.

The analyses were carried out for each of the four contrasts separately. Restricted maximum likelihood (REML) estimation was used to fit the models. All of the analyses were carried out with R and S-Plus, using the metafor [28] and the nlme packages [29].

### Risk of bias across studies

We also examined the presence of publication biases visually (by inspecting the presence of asymmetry in funnel plots) and by including and testing the inverse of the sample size as a potential covariate in the models [30]. A significant relationship between the inverse of the sample size and the observed *d*-values may be suggestive of publication bias.

### Additional analyses

Finally, correlational analyses were employed across all training conditions to investigate whether the change/difference in interpretation bias was associated with changes in negative mood in response to training and in response to an emotional challenge.

To address the potential risk of bias within studies, the main analyses were repeated excluding studies with only one training condition and therefore no random assignment to training condition and excluding conditions that had an attrition rate of >15%.

### Interpretation of Effect Sizes

The standardized mean change values were computed in such a way that more positive (or less negative) values indicate more preferable outcomes (i.e., stronger endorsement of the positive instead of the negative interpretations after the training, stronger endorsement of positive interpretations post- versus pre-training, decreased negative mood post- versus pre-training, and a less pronounced decrease in negative mood post- versus pre-challenge).

Letting φ() denote the cumulative density function of a standard normal distribution, the interpretation of the standardized mean change can also be facilitated by noting that φ(*d*) estimates the proportion of individuals for which the difference or change scores reflect a preferable outcome [31]. For example, an effect size of 0 implies that φ(0) = .50 (i.e., 50%) of individuals should have a larger positive interpretation bias after the training than before (while 50% have a smaller positive interpretation bias) or that 50% of individuals have a decreased negative mood after the training (while 50% have an increase in negative mood). For an effect size of 0.2 (a “small” effect), the positive interpretation bias is expected to increase (and negative mood is expected to decrease) for φ(0.2) = .58 (i.e., 58%) of individuals. Effect sizes of 0.5 (a “medium” effect) and 0.8 (a “large” effect) correspond to an increase in positive interpretation bias (and a decrease in negative mood) in 69% and 79% of individuals, respectively [31].

## Results

### Study selection

**Figure 1:**
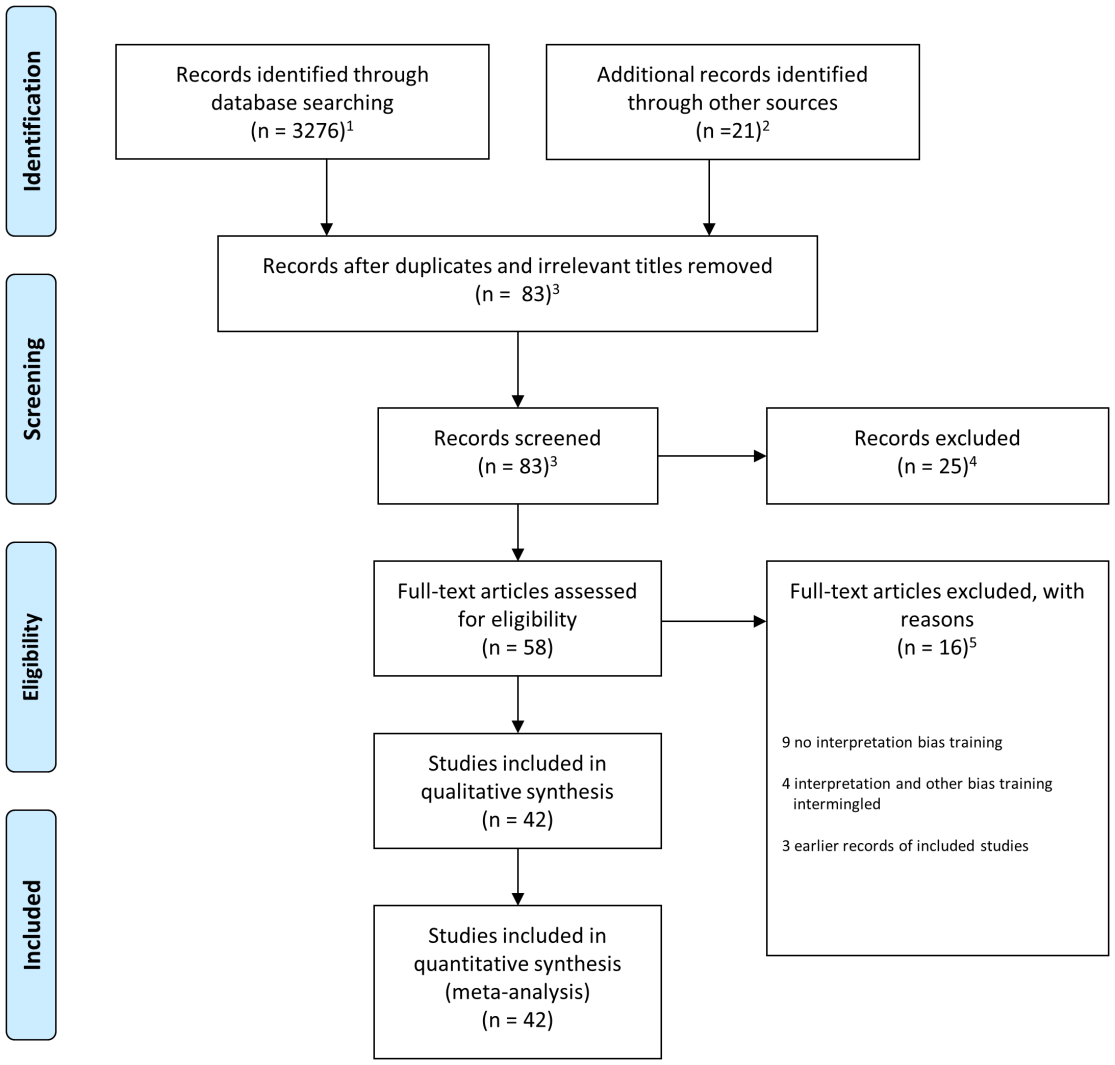
PRISMA flow chart of article retrieval and selection. *Note*. ^1^the search in June 2013 was conducted on separate searches of the data-bases as the software to do simultaneous searches was no longer available. In total, 109 hits were identified in June 2013. ^2^zero records were excluded for the search in 2013. ^3^ten records were screened for the search in 2013. ^4^four articles were excluded for the search in 2013. ^5^three articles were excluded for the search in 2013.

Articles were retrieved according to the PRISMA guidelines [24]. The numbers of articles screened, and included (and reasons for exclusion) can be found in figure 1.

### Study characteristics

All study characteristics are reported in table 1 [5]–[10], [14], [18]–[20], [32]–[63]. In total 42 articles met inclusion criteria (figure 1), of which 28 articles administered the ambiguous situations (AS), six the homograph, four the word-sentence association task (WSAT), and four a combination of two training paradigms or another training paradigm. Combined these articles reported on a total of 59 independent studies (also see table 1). Thirty-eight studies were conducted in healthy participants, and 21 studies in symptomatic individuals. The great majority of studies assessed interpretation bias (*k* = 50) and mood (*k* = 48) as outcome measures, whereas fifteen studies assessed also reactivity to an emotional challenge as an additional outcome measure. Within these studies a total of 125 independent samples were included. Sixty-three samples received a benign, 16 a neutral, 38 negative, and eight no training (for more detail please see table 1). In 80 samples imagery instructions were administered, in 60 samples participants were instructed to generate words or meaning of ambiguity, 94 samples received training in a visual format, 18 in an auditory format, and six samples received a combination of both, and finally, 90 samples received feedback during task administration. In total 2526 individuals were included in the analysis.

### Risk of bias within studies

To address the question whether random assignment and attrition affects results these variables were coded for each study. In total, five included only one training condition and therefore had no random assignment to training group (see also table 1): Blackwell and Holmes [33] trained seven individuals to adopt benign interpretations, Grey and Mathews [5] administered neutral interpretation bias training to 20 individuals, Holmes and colleagues [10] trained 20 individuals to adopt benign interpretations, Mathews and Mackintosh [7] assessed the interpretation bias in 12 individuals in a ‘no training’ condition, and finally Turner & colleagues [62] trained eight individuals to adopt benign interpretations.

Seven independent studies administered interpretation bias training more than once (see also ‘no of sessions (frequency)’ in table 1) and had the following rates of attrition per training condition: Amir and Taylor [19] 15% in the benign and 13.04% in the neutral training condition, Beard and Amir [6] 0% in both the benign and the neutral training condition, Blackwell and Holmes [33] 12.5% in the one benign training condition, Bowler and colleagues [20] 12.29% in the benign and 0% in the no training condition, Lang and colleagues [44] 7.10% in both the benign and the neutral training condition, Mathews and colleagues [48] 0% in both the benign and no training condition, Salemink and colleagues [9] 5.56% in both the benign and neutral training condition, and Williams and colleagues [34] 31.58% in the benign and 12.90% in the no training condition.

### Results of individual studies

**Figure 2:**
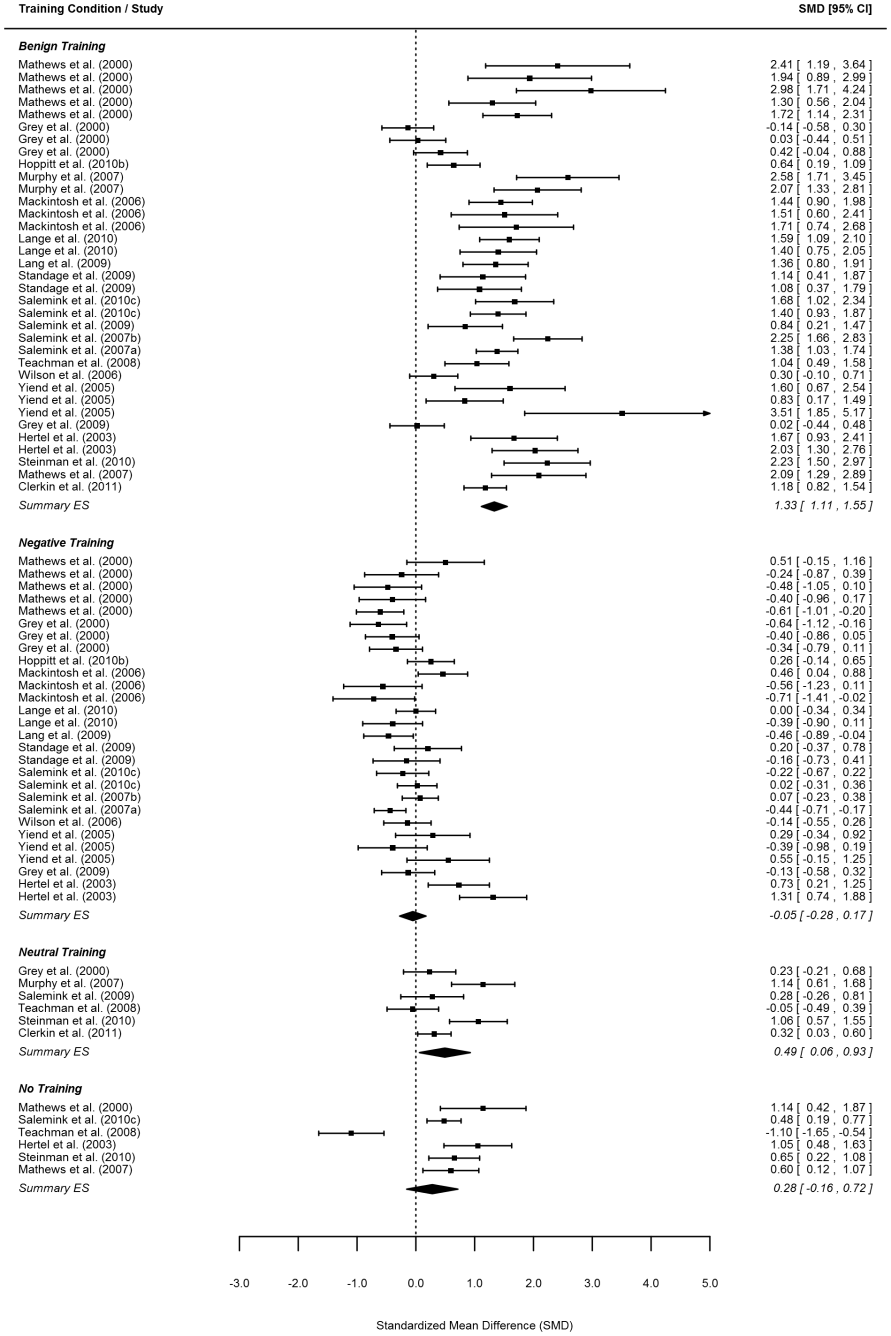
Forest plot of post training difference between positive and negative interpretation bias. *Note*. Order of same conditions within one study follow the order of table 1.

**Figure 3:**
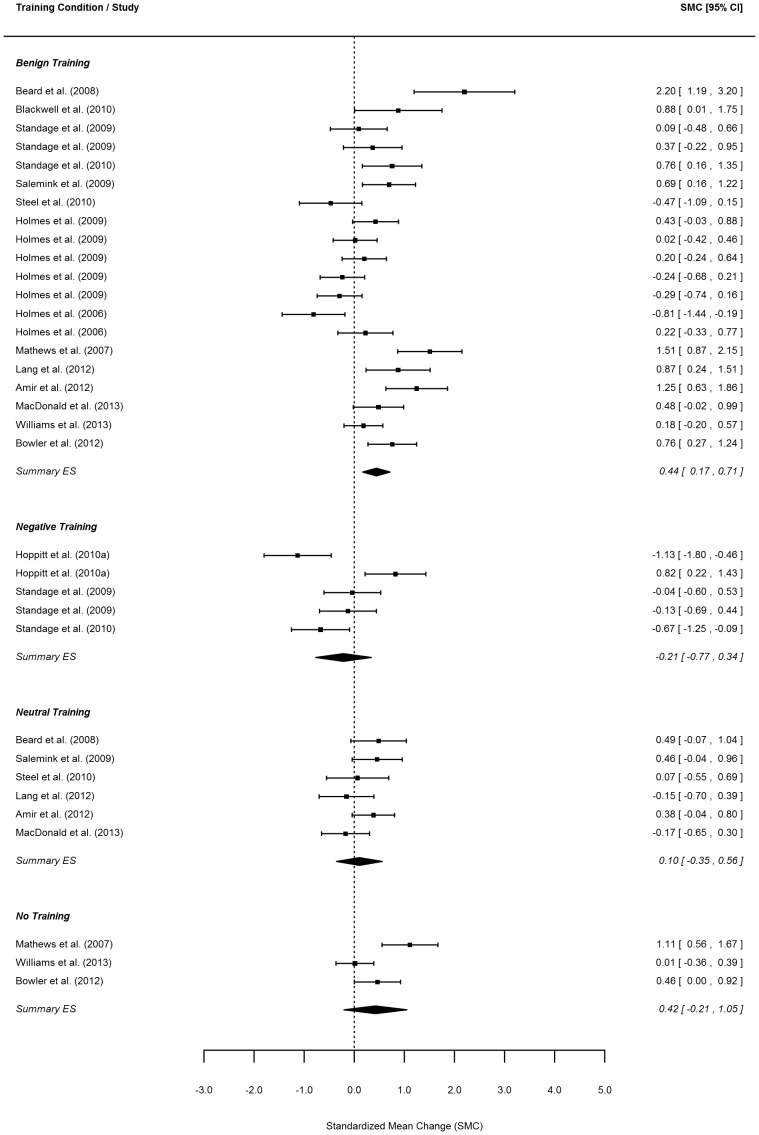
Forest plot of change in positive interpretation bias. *Note*. Order of same conditions within one study follow the order of table 1.

**Figure 4:**
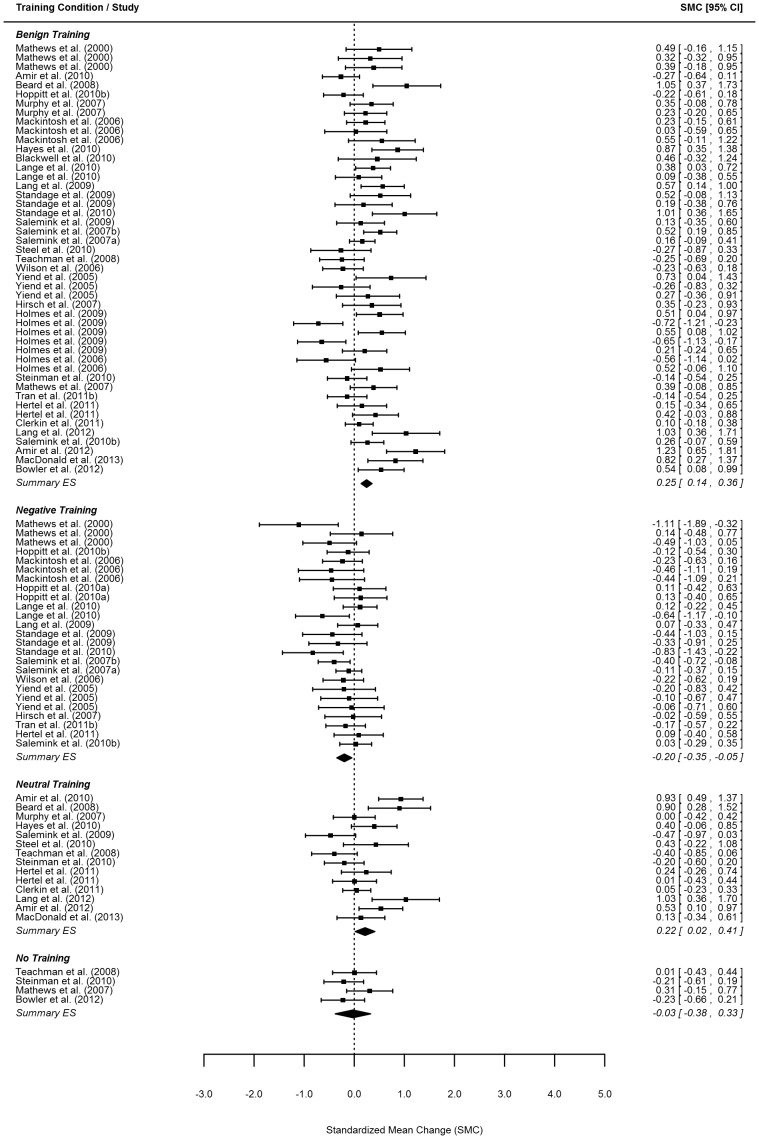
Forest plot of change in negative mood. *Note*. Order of same conditions within one study follow the order of table 1.

**Figure 5:**
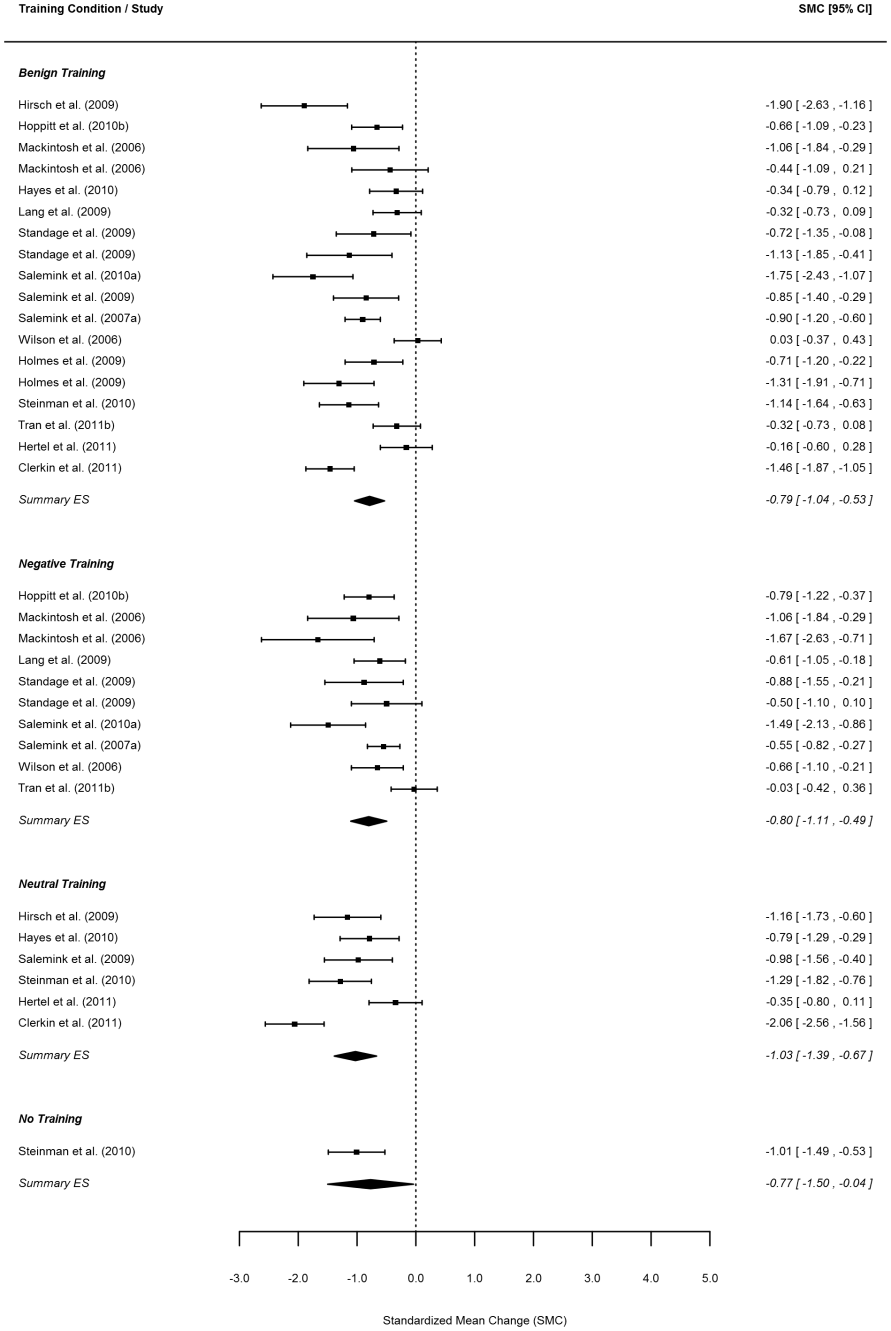
Forest plot of change in negative mood in response to an emotional challenge. *Note*. Order of same conditions within one study follow the order of table 1. The summary effect size for the no-training condition is not identical to the effect size reported for the sole study in this condition as the model took multiple nesting (within study and within one article) into account and ‘corrected’ for such effects in the summary effect size.

Effect size estimates for the change in positive bias and negative mood from before to after training as well as for the change in mood from before to after the emotional challenge are reported per study and training condition in the forest plots in figures 2–5.

### Synthesis of results I: Does benign CBM-I training alter interpretational styles and mood states?

Table 2 reports the pooled within group effects separately for the four training conditions (benign, negative, neutral, no training) on post-training differences in interpretational style (endorsement of positive versus negative interpretations), on pre- to post-training changes in positive interpretation style, on pre- to post-training changes in negative mood state, and on pre- to post-emotional challenge changes in negative mood state. Table 3 contains the pairwise comparisons of the within-group effects across the four training conditions.

**Table 2:** Main results – differences within conditions.

Outcome	K(total)	K(condition)	Trainingcondition	ES	95% CI	*p*
difference between positive and negativeinterpretation bias after training^1^	75	**35**	**Benign**	**1.33**	**1.11; 1.55**	**<.001**
		28	Negative	−0.05	−0.28; 0.17	.66
		**6**	**Neutral**	**0.49**	**0.06; 0.93**	**.03**
		6	No training	0.28	−0.16; 0.86	.72
change in positive interpretation biasfrom pre- to post training^2^	34	**20**	**Benign**	**0.43**	**0.17; 0.69**	**<.01**
		5	Negative	−0.22	−0.75; 0.32	.43
		6	Neutral	0.12	−0.33; 0.57	.59
		3	No training	0.32	−0.30; 0.93	.31
change in negative mood from pre- to posttraining^3^	90	**47**	**Benign**	**0.25**	**0.14; 0.36**	**<.001**
		**25**	**Negative**	−**0.20**	−**0.35;** −**0.05**	**<.01**
		**14**	**Neutral**	**0.22**	**0.02; 0.41**	**.03**
		4	No training	−0.03	−0.38; 0.33	.88
difference in negative mood from pre- to postemotional challenge^4^	35	**18**	**Benign**	−**0.79**	−**1.04;** −**0.53**	**<.001**
		**10**	**Negative**	−**0.80**	−**1.10;** −**0.49**	**<.001**
		**6**	**Neutral**	−**1.03**	−**1.39;** −**0.67**	**<.001**
		**1**	**No training**	−**0.77**	−**1.49;** −**0.05**	**.04**

*Note.* ES = effect size, CI = confidence interval, k = nr of independent samples; ^1^positive values reflect higher positive than negative bias; ^2^positive values reflect increase in positive bias; ^3^positive values reflect decrease in negative mood; ^4^negative values reflect increase in negative mood.

**Table 3:** Main results – differences between conditions.

				Training condition			Omnibus Test
Outcome	K(total)	K(condition)	Trainingcondition	Neutral	Negative	Benign	
difference between positive andnegative interpretation bias after training^1^	75	35	Benign				QM (df = 3) = 137.70, *p*<.01
		28	Negative			1.39 *** (1.15;1.62)	
		6	Neutral		−0.54* (−1.01.;−0.07)	0.84 *** (0.40;1.29)	
		6	No training	0.21 (−0.35;0.77)	−0.33 (−0.79;0.31)	1.05 *** (0.60;1.50)	
change in positive interpretation biasfrom pre- to post training^2^	34	20	Benign				QM (df = 3) = 5.53, *p* = .14
		5	Negative			0.65 * (0.07;1.23)	
		6	Neutral		−0.34 (−1.04;0.36)	0.31 (−0.19;0.81)	
		3	No training	−0.19 (−0.95;0.57)	−0.53 (−1.35;0.28)	0.12 (−0.53;0.76)	
change in negative mood frompre- to post training^3^	90	47	Benign				QM (df = 3) = 24.13, *p*<.001
		25	Negative			0.44 *** (0.26;0.63)	
		14	Neutral		−0.41 ** (−0.66;−0.17)	0.03 (−0.19;0.25)	
		4	No training	0.24 (−0.16;0.65)	−0.17 (−0.56;0.21)	0.27 (−0.10;0.64)	
difference in negative mood frompre- to post emotional challenge^4^	35	18	Benign				QM (df = 3) = 2.11, *p* = .50
		10	Negative			0.01 (−0.25;0.27)	
		6	Neutral		0.23 (−0.17;0.63)	0.24 (−0.09;0.57)	
		1	No training	−0.26 (−0.98;0.46)	−0.03 (−0.77;0.71)	−0.02 (−0.73;0.69)	

*Note.* ES = effect size, CI = confidence interval, * p<.05, ** p<.01, *** p<.001. Results are depicted in such a way that positive numbers indicate a positive change (that is (i) a higher positive than negative bias after training, (ii) an increase in positive bias from pre- to post-training, and (iii) a *de*crease in negative mood from pre- to post-training; ^1^positive values reflect higher positive than negative bias; ^2^positive values reflect increase in positive bias; ^3^positive values reflect decrease in negative mood; ^4^negative values reflect increase in negative mood.

#### Post-training endorsement of positive versus negative interpretations

As shown in Table 2, across 75 independent samples (*k* = 75) (distributed over a total of 34 studies), benign and neutral training demonstrated a significant difference between endorsement of positive and negative interpretations after training. The effect size of this difference for the benign training was substantial (i.e., 1.33 with 95% CI 1.11–1.55). For the same difference in the neutral condition this effect was also significant but considerably smaller (i.e., 0.49, 95% CI 0.06–0.93). This implies that, on average, 91% of individuals who had received benign training and 69% of individuals who had received neutral training endorsed positive interpretations more strongly than negative interpretations for new ambiguous stimuli after training. Importantly, the size of the effect in the benign condition was significantly larger than in the neutral and any other condition using pairwise comparisons of effect sizes (all pairwise *p*<.001; see table 3).

As described earlier, the effect sizes were analyzed with a multilevel model that included a variance component for study level variability (allowing for shifts in the effects across studies irrespective of training condition) and a variance component at the effect size level (accounting for differences in the effects across studies for the various training conditions). Both the study level variance component and the effect size level variance component did reach statistical significance (*p* = .002 and *p*<.001, respectively – not depicted in table 2). Therefore, the findings reported above should be viewed as the *average* effects across the different studies.

#### Pre-post training change in positive interpretation bias

Next, we assessed pre- to post-training changes in the endorsement of positive interpretations for each training condition. Data were available from 34 independent samples (*k* = 34) (from a total of 17 studies). Benign training was the only condition to result in a significant change in the selection of positive interpretations of ambiguous stimuli from before to after training (*p*<.01). This effect was of a much smaller size (i.e., 0.43 with 95% CI 0.17–0.69; table 2) than the one found for post training differences between positive and negative interpretation biases in the benign condition, and can be interpreted as indicating that, on average, 67% of individuals receiving benign training showed an *increase* in the selection of positive interpretations. This change differed significantly (*p*<.05; see table 3 comparing ‘negative’ and ‘benign’ training condition under ‘change in positive interpretation bias from pre- to post training’) from the change in the opposite direction for the negative training condition (i.e., −0.22 with 95% CI −0.75–0.32; table 2). While the effect size level variance component was significantly larger than zero (*p*<.001), we did not find significant study-level heterogeneity (*p* = .58) for this outcome (again, not depicted in table 2). However, since at least one component was significant, this indicates again that the pooled effects need to be viewed as average effect sizes across the different studies.

#### Pre-post training changes in mood state

Data from 90 samples (from 42 studies) were available to assess training effects on changes in negative mood state. Overall negative mood *de*creased significantly (*p*<.001) in the benign training condition (a small effect size of 0.25 with 95% CI 0.14–0.36; table 2) indicating that, on average, 60% of individuals showed reductions in negative mood in the benign condition. In contrast, negative mood *in*creased significantly (*p*<.01) for those receiving negative training (a small effect size of −0.20 with 95% CI −0.35– −0.05; table 2), indicating that, on average, 58% of individuals showed increases in negative mood after negative training (see table 3 under ‘change in negative mood from pre- to post training’). In the neutral training condition negative mood decreased significantly (*p* = .03) from before to after training (*ES* = 0.22; 95% CI 0.02–0.41; table 2). The comparison of the within-group changes *between* the benign and the negative training condition was significant (*p*<.001; table 3). Moreover, while the change in the negative training condition varied significantly from the change in the neutral condition (*p*<.01; table 3), there was no significant difference in the amount of change in mood state between the benign and neutral or no-training conditions (both *p*’s >.05) (see table 3 under ‘change in negative mood from pre- to post training’). Again, only heterogeneity at the effect size level (*p*<.001) and not the study level (*p* = .86) was found suggesting that the pooled effects above need to be viewed as average effect sizes (not depicted in table 2).

#### Pre-post emotional challenge change in mood state

Mood state before and after an emotional challenge was assessed in 35 samples (15 studies). Negative mood increased significantly in all conditions from before to after the emotional challenge (with estimated effects ranging from −0.77 to −1.03, see table 2 under ‘ differences in negative mood from pre- to post emotional challenge’). However, none of the pairwise differences comparing the amount of change between conditions was significant (see table 3 under ‘difference in negative mood from pre- to post emotional challenge’). In contrast to the earlier outcomes, we found significant heterogeneity at the study (*p*<.01), but not at the effect size level (*p* = .18). As before, the pooled effects reflect averages across studies.

### Synthesis of results II: Do training- and sample characteristics enhance the effects of benign training?

Next, we assessed the influence of potential moderators on the effects of *benign* training on the various outcome measures that reached significance for the *within-group* differences (table 2) in our primary analysis. Table 4 shows the influence of the moderator variables on the size of the effects for the different contrasts and their significance (for dichotomous moderators, the values reflect the difference between the two levels of the moderator, for continuous moderators, the values reflect the change in the size of the effect for a one-unit increase in the moderator) based on the meta-regression analyses. Due to the differential number of studies using AS, homograph, WSAT and other paradigms, we were only able to compare the AS task with all other paradigms combined. Additionally, we explored the association between the moderators that revealed significant effects within one outcome measure (except for paradigm as this variable was controlled for in the relevant analyses, also see methods). This was done to inform the interpretation of results as we were only able to assess each moderator separately (see Table S3 for all correlations between moderators per outcome measure).

**Table 4:** Moderators.

	Moderator									
	*Training characteristics*							*Sample characteristics*		
*Outcome*	*training paradigm* *(AS vs other)*	*imagery* *instructions (y/n)*	*word* *generation (y/n)*	*mode of presentation* *(visual/auditory)*	*feedback* *administration (yes/no)*	*Training items/total items (ratio)*	*# sessions* *(frequency)*	*health status (symptoms/* *healthy)*	*mean age*	*% males (sex)*
**cognition**										
difference between positive and negative interpretation bias after *benign* training	0.90 ***	1.05 ^a^ ***	−0.34	0.15	0.06	0.17	n.a^b^	0.30	−0.00	−0.02 *
change in positive interpretation bias from pre- to post *benign* training	−0.95 ***	0.50 **	n.a ^c^	−0.50	0.46 *	−2.78 **	0.08 **	0.69 **	−0.02	−0.03 **
**mood**										
change in negative mood frompre- to post *benign* training	−**0.29** ^d^ *	0.50 **	Interaction with paradigm^e^	−0.14	0.20	−0.34	0.07 *	0.15	−0.00	−0.01 **
**emotional challenge**										
change in negative mood from pre- to post emotional challenge	0.23	−0.07	0.16	−0.15	0.05	0.46	−0.01	−0.20	0.01	0.02 **

*Note.* Regression coefficients of the meta-regression models including the respective moderator. If paradigm resulted in a significant moderation of training effect, it was included in the tests of the other training-characteristic variables. *p<.05, ** p<.01, ***p<.001; ^a^perfect confounding with paradigm; ^b^ there were 33 samples with one training session, one sample with four, and one sample with eight training sessions, it could thus not reasonably be tested; ^c^ there were only two samples asking individuals to generate word fragments and 15 that exposed participants to training stimuli, therefore no reasonable testing was possible; ^d^ *p* = .05; ^e^ there was a significant interaction with paradigm (*β* = −.75, *p* = .02) indicating that the effect of generation depends on the paradigms used, for all combined other paradigms (i.e. homograph, WSAT, or other; *k* = 6) the effectiveness is reduced when generation is employed as compared to when no generation is employed, whereas this is not true for the AS paradigms.

#### Post-training endorsement of positive versus negative interpretations

The paradigm employed significantly impacted on the post-training difference between positive and negative interpretation bias in that the AS paradigm (*k* = 26) was significantly more effective (Table 4). All other paradigms combined (*k* = 9) yielded a significant effect size of.69 (*p*<.001), indicating that there is a 75% chance (on average) for endorsing positive interpretations more strongly than negative interpretations. For AS-paradigms this effect increased by ES = .90 (see table 4), yielding an overall effect of.69+.90 = 1.59, which corresponds to a 94% chance for endorsing positive interpretations more strongly than negative interpretations. In absolute terms, that is a 94%−75% = 19% percentage points difference. Relative, that is 94/75 = 1.25, that is a 25% higher chance of endorsing positive interpretations more strongly than negative interpretations. Similar findings emerged for the use of imagery instructions. No use of imagery instructions (*k* = 8) resulted in an effect size of.54 (*p*<.01) indicating a chance of about 71% for endorsing more positive than negative interpretations. This effect increased by *ES* = 1.05 (*p*<.001), when imagery instructions were employed (*k* = 27)(see Table 4), resulting in an overall ES of 1.59 indicating a chance of almost 94% of endorsing more positive than negative interpretations, that is a 33% higher chance than when no imagery instructions were employed.

However, it needs to be noted that paradigm and imagery-use were almost perfectly confounded; all AS-paradigms employed imagery instructions, whereas only one of the nine homograph paradigms did. It is therefore impossible to tell whether paradigm or the use of imagery is driving these results. Finally, while in all-female samples the difference between positive and negative interpretation biases was large and significant (*ES* = 2.03, *p*<.001, 98% chance of endorsing more positive than negative interpretations after training), this difference decreased by *ES* = −.02 (*p*<.05) per percentage point more males in the sample (see table 4). Thus, in a sample with for example 40% males this converges to a total effect of 1.28, corresponding to an absolute chance of 90%, which is a 9% decreased chance of endorsing more positive than negative interpretations after training.

For the endorsement of positive versus negative interpretations after training, the use of imagery instructions and male sex within the sample correlated by *r* = −.60 indicating that samples including fewer male participants were more often instructed to use imagery.

#### Pre-post training change in positive interpretation bias

For the change in positive interpretation bias from pre- to post-training, again paradigm, imagery instructions, and sex emerged as significant moderators.

However, this time the AS-paradigm (*k* = 14) yielded a non-significant increase in positive interpretations (*ES* = .14, *p* = .26, 56% chance of an increase in positive interpretation bias). This effect was then, however, significantly increased by *ES* = .95, *p*<.001) when ‘other’ paradigms (*k* = 6; 1 homograph; 3 WSAT, and 2 other paradigms) were employed. This results in an overall *ES* = 1.08 corresponding to a 54% higher chance of an increase in positive interpretation bias. In line with the findings for the difference between positive and negative interpretations after training, again, imagery instructions yielded a significant effect. Whereas no use of imagery instructions (*k* = 6) did not yield a significant effect (*ES* = −.20, *p* = .21, 42% chance of increase in positive interpretation bias), this effect was significantly enhanced by *ES* = .50 (*p*<.01) when imagery instructions were employed (*k* = 12). This results in a net-effect of imagery instructions of *ES* = .30 (62% absolute chance and 48% higher chance of increase in positive interpretation bias). Additionally, while no use of feedback (*k* = 12) yielded a non-significant small effect (*ES* = .01, *p* = .96, corresponding to a 54% chance for an increase in positive interpretations), the use of feedback (*k* = 8) increased this effect significantly by *ES* = .46 (*p*<.05). This results in a total effect of feedback administration of *ES* = .46 (68% absolute chance and 26% higher chance of increase in positive interpretation bias). Also, the ratio of benign training items versus all training items (ratio 0.69 (*k* = 2), 0.80 (*k* = 1), 1.00 (*k* = 17)) showed a significant effect. While the ratio of.69 yielded a large effect (*ES* = 0.94, corresponding to a 83% chance of an increase in interpretation bias) this effect was significantly reduced when the ratio became 1.00 (*p*<.01). This results in an absolute effect of *ES* = .07 (53% absolute chance and 57% lower chance of increase in positive interpretation bias). However, as only three samples employed a ratio <1, this result needs to be viewed with caution. Finally, adding one training session significantly increased the effect of training by *ES* = .08 (*k*_one session_ = 12; *k*_four sessions_ = 2; *k*_six sessions_ = 1; *k*_seven sessions_ = 2; *k*_eight sessions_ = 2; *k*_twelve sessions_ = 1, *p*<.01; total *ES* = .01; absolute chance of 51% and higher chance of 6%). Although this may seem a very small effect, the effect of 10 sessions of training would already result in an *ES* = .80, with a higher chance of approximately 49%. Exploring the potential clinical benefits of benign CBM-I, it was particularly interesting to see that while the effect for healthy samples was non-significant (*k* = 10, *ES* = .07, *p* = .65, c.a. 53% chance of increase), this effect increased by *ES* = .69 (*p*<.01) in samples with mood-symptoms (*k* = 10) resulting in a total effect of *ES* = .76, absolute chance of 78% and higher chance of 47% of increase in positive bias. Conform above results, while all-female samples showed a large and significant effect (*ES* = 1.30, *p*<.001, 90% chance of increase), this effect was significantly reduced by *ES* = .03 (*p*<.01) per percentage point males in the sample. Thus, a sample with for example 40% males, the chance for an increase in positive interpretation bias would be lowered by 61%.

None of the moderators demonstrating a significant effect correlated strongly with another (all *r’*s ≤.50), except for the number of training sessions and health status of individuals (*r* = .73) (also see Table S3). This indicates that particularly samples with symptoms of depression and/or anxiety were exposed to a repeated number of training sessions. Post-hoc analyses including both ‘number of training sessions’ and ‘health status’ revealed the following effects: *ES* = .09, *p*<.05 for ‘number of sessions’ and *ES* = −.01, *p* = .99, for ‘health status’ suggesting that the effect for ‘no of sessions’ depicts a rather robust effect. However, it is important to mention that more than one training session was only administered to individuals with symptoms whereas one training session was administered to both healthy and symptomatic samples (also see table 1) making it difficult to meaningfully interpret this result.

#### Changes in mood state

The change in negative mood from pre- to post-training in benign training conditions was again moderated by paradigm, imagery, and sex.

The AS paradigm resulted in a significant decrease in negative mood (k = 37; *ES* = .19, *p*<.01, 58% chance of decrease in negative mood). This effect was significantly increased by *ES* = .29 (*p* = .05) when other paradigms were employed (*k* = 10). The total effect when other paradigms were employed therefore was *ES* = .48, with an absolute chance of 68% and a higher chance of 17% of decrease in negative mood. The use of no imagery instructions resulted in a non-significant effect (k = 11; *ES* = −0.25, *p* = .083, 40% chance of decrease in negative mood). However, adding imagery instructions (*k* = 33) increased this effect significantly by *ES* = .50 (*p*<.01), resulting in a total *ES* = .25, which corresponds to a 60% absolute and 50% higher chance of decrease in negative mood. Adding one session (*k*_one session_ = 40; *k*_four sessions_ = 2; *k*_six sessions_ = 1; *k*_seven sessions_ = 2; *k*_eight sessions_ = 2; *k*_twelve sessions_ = 1) resulted in a significant larger decrease in negative mood by increasing the effect by *ES* = .07, *p*<.05, resulting in a total *ES* = .16, which corresponds to a 56% absolute and 4% higher chance of decrease in negative mood when administering for example two instead of one session. Finally, while all-female samples showed a significant decrease in negative mood (*ES* = .70, *p*<.001, 76% chance), this effect was significantly decreased per one percentage male by *ES* = −.01, *p*<.01. Thus, for 40% males in the sample, the total ES would reduce to ES = .30, corresponding to a 23% lower chance. The correlations between the significant moderators here were small (all *r*’s <.36) (also see Table S3).

To sum up, training paradigm, imagery instructions, the number of training sessions, and sex moderated the impact of benign training on at least two different outcome measures. Feedback administration, the ratio between benign training items and the total number of training items, and health status moderated the impact of benign training on the change in positive interpretation bias from pre- to post training only. Although most correlations between the significant moderators were far from perfect (≤.5), the effects of repeated number of training sessions and mood symptoms for the increase in positive interpretation bias were strongly associated and therefore need to be viewed with caution.

### Risk of bias across studies

The *p*-values of the tests for funnel plot asymmetry for the four outcomes were.72,.79,.46, and.89, respectively. Therefore, based on these tests and the visual examination of the funnel plots (Figure S1), there was no indication of publication bias for any of the main outcomes, as indicated by the absence of an association between the inverse of the sample sizes and the effect sizes [30] (see Figure S1 for some additional analyses possibly hinting at some asymmetry in the pre-training versus post-training endorsements of positive interpretations outcome).

### Additional analyses I: Do training effects on interpretation bias and on mood state correlate?

Based on *k* = 49 pairs of effect size estimates, the correlation between the endorsement of positive versus negative bias after training and the decrease in negative mood was positive (*r* = .60; CI 0.39–0.76; *p*<.001). The correlation between the change in positive interpretation bias from before to after training and decrease in negative mood was also positive (*r* = .58; CI 0.30–0.78; *k* = 32 pairs of estimates; *p*<.001). There was no significant correlation between the endorsement of positive versus negative bias after training or the change in positive bias from before to after training and the increase in negative mood in response to an emotional challenge (*r* = −.0001, CI −0.41–0.41, *k* = 23 and *r* = −.15, CI −0.77–0.62, *k* = 8, respectively).

### Additional analyses II: Does randomization and percentage of attrition affect main results?

Excluding all studies with only one training condition (and therefore no random training allocation) and training conditions with an attrition rate above 15% did not change the main results.

## Discussion

Cognitive bias modification training for interpretation bias (CBM-I) has recently been considered a promising clinical tool, e.g., see [33] with potential for boosting positive thoughts (i.e., positive or benign interpretations of ambiguous situations) and thereby improving emotional symptoms. The current meta-analysis addressed some outstanding questions of relevance for the potential use CBM-I in a clinical setting.

### Does benign CBM-I boost positive interpretations with improvements in negative mood?

In the current analyses, benign CBM-I resulted in large post-training endorsements of positive relative to negative interpretations, in small to medium changes in positive interpretational style from pre- to post training, and small decreases in negative mood states from pre- to post training. The correlation between these indices of improvements (i.e., change in interpretational style and mood state) was positive and significant. While these benign training effects differed reliably from negative training effects, the difference relative to neutral or no-training conditions remained insignificant for the change in positive bias and mood. Furthermore, benign training did *not* attenuate relative increases in negative mood in response to emotional challenges.

These findings partly support but also extend results from the earlier meta-analysis which combined assessment of CBM-I effects with another training program, CBM-A [11]. Across both meta-analyses (the current one including additional 24 articles) benign CBM-I training reliably ‘boosted’ positive interpretations post-training. Benign CBM-I resulted in a large post-training difference *between* positive and negative interpretation bias, which was also significantly larger than in any comparison condition. However, the neutral condition also presented with a significantly larger positive as compared to negative interpretation bias after training. As these effects are based on post-training differences only, it is impossible to tell whether the *change* in positive interpretation bias due to training was significant in the benign as well as in the neutral group. We therefore additionally systematically investigated the increase in positive interpretation bias from pre- to post-training. The benign condition showed significant but small changes, which differed from negative training only. This raises the question of whether benign training significantly enhances changes that are attributable to naturally occurring fluctuations in interpretational style (as those demonstrated by individuals receiving no training) and changes that may be explained by placebo effects (as those demonstrated by individuals receiving neutral training). However, it also needs to be taken into account that compared to the benign training no-training and neutral training were administered less frequently (three and six samples as compared to 20 samples), hence power for these comparisons might have been reduced. Including these comparative training conditions in primary studies might address these questions more reliably in the future. Yet, it is still notable that only benign training showed a significant *change* in interpretational style, indicating its potential as an interventive tool.

Whereas benign CBM-I seems to be the only condition to significantly affect interpretational biases, mood was affected in all but the no-training condition. Benign CBM-I training resulted in significant, but small decreases in negative mood, which significantly differed from changes in the opposite direction in the negative condition, but not from changes in neutral and no-training conditions. The significant positive change in the neutral condition but not in the no-training condition may suggest that neutral training is not completely emotionally ‘neutral’ after all. These findings may help to explain the inconsistencies in the primary literature, e.g. see [64], as it shows that the significance of the post training differences in mood probably only becomes obvious when benign and negative groups are compared.

Furthermore, contrary to predictions that the tendency to benignly interpret ambiguous situations should decrease the negative mood response particularly under conditions of emotional provocation [63], we did not find this in the present data. To understand what this implies it is crucial to inspect the diverse emotional challenges employed to investigate negative mood reactivity after CBM-I. Those included overall stressful videos [8], [39], [43], [45], [63], unsolvable anagram tasks [9], [52], worry intrusions or negative mood inductions [10], [37], [40], [50], symptom provoking tasks [35], [58], speech anticipation [55], and exposure to emotional faces combined with ‘incorrect’ feedback [61]. It can be suggested that most of those did not provoke ambiguity specifically, but were distressing more generally. For example, watching stressful videos [39], trying to solve an unsolvable anagram task under time pressure ‘knowing’ that ‘most people have no problems solving it in time’, e.g., see [9] or giving a speech, e.g. see [55] should be stressful for most individuals. The results of the current meta-analysis therefore only suggest that benign CBM-I does not alter overall mood-reactivity in response to these universally stressful events or situations. Very likely, emotional challenges currently employed were arguably not the most suitable for assessment of the more subtle effects of interpretation biases on negative mood-reactivity as the reaction to these stressors are not under the direct influence of interpretation biases. Future studies should consider including emotional challenges that more directly activate the manipulated cognitive mechanism allowing clinically relevant conclusions about benign CBM-I’s effects on daily-life stressors, such as having a group of colleagues laughing when you enter the room.

Nevertheless, the current data support the hypothesis that trained differences in interpretational style and changes in mood state are correlated, implying that benign interpretation biases are related with feeling less negative. The manipulation of interpretation style not only resulted in significant *changes* in interpretation style, but these changes were also significantly associated with decreases in negative mood states. Although these mood changes appeared to be of rather small effect size, assuming that individuals are confronted with ambiguous situations repeatedly in their daily lives, the cumulative effect of benignly interpreting these situations might result in clinically significant improvements in the long run.

### How can we boost benign CBM-I’s effectiveness?

To amplify these positive effects of benign CBM-I, it is of upmost clinical relevance to know *how* training effects can be maximized. The probably most obvious factor, training paradigm, showed somewhat unexpected findings. We distinguished between two paradigm groups: the ambiguous situations paradigm (AS) and the combination of all other employed tasks. While the chances for a larger positive than negative interpretation bias after training were higher for individuals receiving the ambiguous situations paradigm (AS), the chances for an increase in positive interpretation bias from pre-to post training were larger for individuals receiving other training paradigms. A possible explanation is that the assessment of the post-training endorsement of positive versus negative bias (but not of the pre-post training differences in positive interpretation bias) shows great similarity to the training method employed in AS-paradigms whereas studies investigating changes in positive interpretations typically use questionnaire measures that do not resemble the employed training program. In support of this suggestion, previous research has shown that, while the endorsement of positive versus negative interpretation style after training showed significant training effects with the AS-paradigm, other interpretation bias assessment tools did not [53]. So, perhaps training effects are more visible when training and test are of increased similarity. Alternatively, the findings for the post-training endorsement of positive versus negative bias may have been solely driven by the use of imagery instructions which were (almost) only employed in studies with the AS-paradigm. From the current results it impossible to tell whether the AS-paradigm would still be superior to the homograph paradigm if no imagery instructions had been employed. Overall, the AS-paradigm was developed to increase stimuli realism [7], [64] and is a task with high face validity. In order to overcome this methodological issue, future studies should consider adding different outcome measures.

Despite this imprecision about which paradigm provides the best results, several procedural factors were found to significantly increase benign CBM-I’s effectiveness *above* and *beyond the type of* training paradigm. Most convincingly, imagery instructions were able to significantly boost improvements in both positive interpretation bias and negative mood. Imagery has long been assumed to have a special link with emotions [18]. The use of imagery in CBM-I has been proposed to increase training effects and primary studies have supported this idea [10], [18]. This is the first meta-analysis indicating that imagery instructions can have moderate to large effects on increasing the effect of benign CBM-I on interpretation biases and accompanying mood. It needs to be considered however, that the use of imagery was associated with percentage females in the sample for post training endorsement of positive versus negative interpretations. This may indicate that the effect of imagery for this particular outcome is partly attributable to the effect of sex and vice versa. However, both sex and imagery yielded significant effects for each of the other outcome measures as well (where only very weak correlations were observable) supporting the robustness of the imaging effect. We therefore suggest that the general finding of the value of imagery instructions in benign CBM-I is valid.

Likewise, the observation that provision of repeated training sessions increased training effectiveness further confirms theory and findings of primary studies focusing on the clinical use of CBM-I. Although the effect sizes were small (.08 for increase in positive interpretation bias and.07 for decrease in negative mood), it is important to realize that this is the increase in effect size for just adding *one* additional training session. Therefore, this effect increases to a certain degree with additional training sessions. However, it is crucial to understand here that repeated training sessions were most often employed in samples with mood-symptoms (with the latter also showing significant influence on the increase in positive interpretation bias). This makes it difficult to determine which of the two moderators drove this effect although post-hoc analyses including both factors simultaneously supported the robustness of the influence of more training sessions. In the future, using internet based CBM applications in combination with momentary assessment techniques [65], [66] may enhance the feasibility of having people ‘train’ their positive interpretation biases more frequently. For example, people may download a simple training program and carry out exercises on a weekly or daily basis, thereby integrating positive interpretation tendencies to their daily life situations. One pioneering study [34] already demonstrated the feasibility and effectiveness of a multi-session internet-based CBM-I. While not influencing any of the other outcome measures, administering feedback about response accuracy and having a moderate ratio of benign training items and total items within the training was further related to a larger increase in positive interpretation bias with moderate (feedback) to large (ratio) effect sizes. Speculatively, both these factors foster engagement with the training task. That is, feedback is only administered if participants have to respond in some way or another to the stimuli presented (not necessarily by completing word-fragments as was assessed with the ‘generate’ variable that showed no effect) and may encourage them to concentrate and elaborate on the information presented. Having not 100% of the items benign may have a similar effect. It has been shown in studies of instrumental conditioning that responses learned under partial reinforcement are much harder to extinguish than responses learned under continuous reinforcement [67]. The same may be true for the current task where participants had to learn on most but not all occasions to interpret situations as benign – which is presumably also somewhat more reflective of real-life interactions [48].

### Do people who are most vulnerable actually respond to CBM-I?

We also sought to examine whether benign CBM-I was universally effective, or if it was of particular benefit to those who were most vulnerable. Our findings indicated that particularly women (who are more vulnerable to develop mood symptoms) tend to benefit more from benign CBM-I, both cognitively (interpretation bias) and emotionally (mood) with significant and large effect sizes. This is in agreement with theory, as it has long been recognized that women are more emotionally reactive than men, e.g., see [21].

The increase in positive interpretation bias from pre- to post-training was particularly prominent in samples with anxiety and mood symptoms/diagnoses as compared to healthy (non-symptomatic) controls (showing a non-significant change). However, as already mentioned above, this effect of mood-symptoms cannot completely be torn apart from the effect of repeated training sessions. It is possible that both effects add a significant part but are smaller than suggested here. Although post-hoc analyses did not support the influence of emotional symptoms, it is important to notice that all studies administering more than one training session were conducted in symptomatic samples, possibly suggesting that repeated sessions are of added value within symptomatic samples. To resolve this issue, research administering repeated sessions to both healthy and symptomatic samples are needed. Exposure to one or more sessions of benign CBM-I may result in only moderate changes in healthy populations as they already possess a substantial positive interpretation bias to begin with, resulting in a ceiling effect. However, for people with a potentially lowered positive interpretation bias, benign CBM-I can cause a significant and large increase in positive interpretation bias. Therefore, benign CBM-I may add therapeutic benefit to general Cognitive Behavioral Therapy (CBT) paradigms by targeting dysfunctional cognitions more directly and specifically without much additional effort from both the therapist and the patient. Very recently, two studies intriguingly demonstrated that benign CBM-I was indeed associated with clinical relevant improvements [20], [34].

### Limitations and future directions

Several issues need consideration in interpreting the current findings. First, a substantial threat to conclusive meta-analyses is the problem of unpublished data. Often, studies that yield significant effect-sizes are more likely to be published than studies not yielding significant effect sizes, a phenomenon referred to as publication bias [30]. To draw firm conclusions about the effectiveness of CBM-I, it is necessary to include as many studies as possible (preferably all). To this end, we contacted all authors in the field to request unpublished data, and also verified that any poster-abstract findings were included in the analysis. Moreover, as there was no indication for publication bias [31], we cautiously suggest that even had there been other unpublished data, these would not have changed the results.

Second, in the current meta-analysis we specifically focused on mood state in order to be as precise as possible in what CBM-I actually does, based on what has actually been assessed. Low mood is a common symptom of mood- and anxiety disorders [15]. However, it does not encompass the whole spectrum of possible mood symptoms. Based on the current findings, it may be concluded that the symptom of ‘low mood’ can be influenced by benign CBM-I. However, this effect is small and whether other symptoms like feelings of guilt or rumination can also be influenced cannot be answered. It needs to be considered though that CBM-I effects on general psychopathology may not immediately become apparent after a single session of CBM-I training for two reasons. First, interpretation biases only start to play a role in reducing mood when people are confronted with ambiguous situations in their own life. Second, interpretation biases represent only one potential bias in information processing [64], which, in turn, represents only one possible cause of emotional disturbance [11]. Studies are currently emerging showing that training more than one benign cognitive bias (e.g., interpretation and attention bias) [68], [69] and repeated training sessions of CBM-I [20], [34] result in clinical relevant improvements.

Finally, it needs to be acknowledged that the current moderators were all investigated separately, preventing conclusions about the sum of their effects when included simultaneously. We did control all analyses for the paradigm employed as this was a rather complex construct encompassing stimulus presentation and processing. Therefore, the current results depict the effects of the moderators above and beyond the effect of paradigm but not above and beyond the presence of any other moderators. However, testing the effects of all possible moderators to investigate their combined additive and interactive effects would have drastically reduced the power to draw any meaningful conclusions. Most of the correlations between the moderators were small to moderate reducing the likelihood that the results were confounded by collinearity. However, - and as already elaborated above -, the effect of repeated training sessions and mood symptoms on the increase in positive interpretation bias in benign training conditions remains indistinguishable so far. Although it needs to be noted that both are intuitively valid and have been supported by primary research, the currently found effects may depict overestimations. In the future, the advent of internet access and momentary assessment technology, like currently developed apps for smartphones, can be used to implement benign CBM-I more efficiently. Participants can download programs to their private computers and train more frequently than possible in the lab. Having a training program on their smartphones would even enable participants to train benign interpretations in their daily lives. Because imagery seems to play a crucial role in increasing benign CBM-I effectiveness, it might be particularly interesting to examine whether novel technical tools such as virtual reality can enhance the computerized training even more. Furthermore, as adolescents are particularly vulnerable to emotional disturbance [70] and initial primary studies have demonstrated effectiveness of CBM-I in these samples [71]–[73], future research needs to demonstrate whether the current results for adult samples translate to adolescent samples.

### Conclusions

Although results were less strong as previously suggested, it may be concluded that benign CBM-I has clinical potential. It was associated with an increase in positive interpretation bias and a decrease in negative mood. These effects (i.e., increase in positive interpretation bias and decrease in negative mood) were significantly correlated indicating that an improvement in cognitive style goes hand in hand with immediate improvements in mood. Although effects did not consistently differ from control training conditions and the effects of repeated number of training sessions was not well distinguishable from the effects of mood-symptoms for the change in positive interpretation bias, employing imagery instructions in particular and to a lesser extent repeated training sessions, a moderate ratio of benign training items and total items, and administering feedback during training can significantly increase these effects. Most encouragingly, there are indications that benign CBM-I paradigms appear particularly effective in vulnerable samples.

## Supporting Information

Figure S1 **Funnel Plots for All Condition Level Effect Sizes and for All Possible Pairwise Differences Effect Sizes.**
(DOC)

Table S1 **PRISMA Checklist.**
(DOC)

Table S2 **Order of single measurements per outcome category.**
(DOCX)

Table S3 **Correlations between moderators per outcome measurement.** *Note.* IMAGE = imagery instructions (yes/no); GENERATE = participants had to generate disambiguating words (yes/no); PRESENT = stimulus presentation (visual/auditory); RATIO = ratio of items in benign direction relative to all items; FREQ = number of training sessions; STATUS = health status (healthy/symptomatic); AGE = mean age of sample in years; SEX = male/female.
(DOCX)
