# Supplementary material for: How to Boost Positive Interpretations? A Meta-Analysis of the Effectiveness of Cognitive Bias Modification for Interpretation
Source: PLoS One. 2014 Jun 26;9(6):e100925. doi: 10.1371/journal.pone.0100925 (PMC4072710; doi:10.1371/journal.pone.0100925)
Supplement: Figure S1 — Funnel Plots for All Condition Level Effect Sizes and for All Possible Pairwise Differences Effect Sizes. (DOC) [file pone.0100925.s001.doc]

**S4 Funnel Plots for All Condition Level Effect Sizes**

**Note:** The *p*-values for funnel ploy asymmetry reported in the paper correspond to the data shown in the funnel plots above. These plots/analyses are the based on the condition level effect sizes (i.e., the standardized mean differences for positive versus negative endorsements or the standardized mean changes between the pre- and post-training/emotional challenge assessments within each condition).

**Funnel Plots for All Possible Pairwise Differences Effect Sizes**

**Note:** The figures above are based on *contrasts* of the set of non-redundant comparisons within studies (e.g., if a study examined three different types of conditions, then this leads to two non-redundant pairwise contrasts – with one condition serving as the reference condition). The x-axis therefore corresponds to the difference in d values for a particular comparison of two conditions, while the y-axis corresponds to the sum of the group sizes of the two groups/conditions being compared. Due to the repeated use of the reference condition in computing these effects, the data are not independent. An appropriate analysis of such data therefore requires a ‘contrast-based’ model as described by Salanti et al. (2008) with appropriately computed covariances for the sampling errors. Examining these data for a relationship between the effect sizes and the inverse sample sizes with such contrast-based models leads to p-values of .37, .05, .17, and .75, respectively. Therefore, for the pre-training versus post-training endorsements of positive interpretations outcome (top right), there may be a hint of asymmetry, but it is unclear on which side of the funnel plot suppression of effects may have occurred.
