# Supplementary material for: How to Boost Positive Interpretations? A Meta-Analysis of the Effectiveness of Cognitive Bias Modification for Interpretation
Source: PLoS One. 2014 Jun 26;9(6):e100925. doi: 10.1371/journal.pone.0100925 (PMC4072710; doi:10.1371/journal.pone.0100925)
Supplement: Table S2 — Order of single measurements per outcome category. (DOCX) [file pone.0100925.s003.docx]

**S2 Order of single measurements per outcome category**

Order was determined based on measured concept, face validity of measurement tool employed, and frequency of usage in collection of primary studies

**COGNITION MEASUREMENTS ORDER**

| **ORDER** | **CODE** | **NAME** |
| --- | --- | --- |
| 1 | C01 | Recognition ratings |
| 2 | C02 | RT to word fragments |
| 3 | C03 | Word sentence association task |
| 4 | C04 | Lexical decision task |
| 5 | C14 | Emotionality ratings, ambiguous description |
| 6 | C06 | Scrambled sentence test |
| 7 | C05 | Form and describe image |
| 8 | C07 | Interpretation bias questionnaire |
| 9 | C13 | Video ratings |
| 10 | C12 | Reason for events |
| 11 | C18 | BBSIQ (Bodily sensation symptoms) |
| 12 | C17 | VAS depressive bias |
| 13 | C19 | RIP (response to intrusions questionnaire) |

**MOOD MEASUREMENT ORDER**

| **ORDER** | **CODE** | **NAME** |
| --- | --- | --- |
| 1 | M01 | STAI-state |
| 2 | M02 | STAI-trait |
| 3 | M06 | NAS (negative affect scale, PANAS) |
| 4 | M08 | VAS anxiety |
| 5 | M09 | VAS depression |
| 6 | M10 | FNES (fear of negative evaluations scale) |
| 7 | M21 | PANAS FS (PANAS fear subscale) |
| 8 | M13 | Libowitz Social Anxiety Scale |
| 9 | M22 | Anxiety Sensitivity Index |
| 10 | M11 | SPAI (social phobia and anxiety inventory) |
| 11 | M20 | VAS sad |
| 12 | M27 | VAS negative |
