# Supplementary material for: How to Boost Positive Interpretations? A Meta-Analysis of the Effectiveness of Cognitive Bias Modification for Interpretation
Source: PLoS One. 2014 Jun 26;9(6):e100925. doi: 10.1371/journal.pone.0100925 (PMC4072710; doi:10.1371/journal.pone.0100925)
Supplement: Table S3 — Correlations between moderators per outcome measurement. Note. IMAGE = imagery instructions (yes/no); GENERATE = participants had to generate disambiguating words (yes/no); PRESENT = stimulus presentation (visual/auditory); RATIO = ratio of items in benign direction relative to all items; FREQ = number of training sessions; STATUS = health status (healthy/symptomatic); AGE = mean age of sample in years; SEX = male/female. (DOCX) [file pone.0100925.s004.docx]

| **S 3 Correlations between moderators per outcome measure** | | | | | | |  |  |  |
| --- | --- | --- | --- | --- | --- | --- | --- | --- | --- |
|  |  |  |  |  |  |  |  |  |  |
| **1) difference between positive and negative interpretation bias after training** | | | | | | | |  |  |
|  | IMAGE | GENERATE | PRESENT | FEEDBACK | RATIO | FREQ | STATUS | AGE | SEX |
| IMAGE | 1.00 | 0.12 | 0.20 | 0.31 | 0.56 | 0.12 | 0.32 | -0.19 | -0.60 |
| GENERATE | 0.12 | 1.00 | -0.46 | -0.15 | 0.02 | 0.05 | 0.08 | -0.22 | 0.03 |
| PRESENT | 0.20 | -0.46 | 1.00 | 0.06 | 0.39 | -0.08 | 0.20 | -0.20 | -0.19 |
| FEEDBACK | 0.31 | -0.15 | 0.06 | 1.00 | -0.22 | 0.04 | 0.10 | -0.45 | -0.18 |
| RATIO | 0.56 | 0.02 | 0.39 | -0.22 | 1.00 | -0.03 | 0.13 | 0.29 | -0.07 |
| FREQ | 0.12 | 0.05 | -0.08 | 0.04 | -0.03 | 1.00 | 0.39 | 0.06 | -0.20 |
| STATUS | 0.32 | 0.08 | 0.20 | 0.10 | 0.13 | 0.39 | 1.00 | -0.30 | -0.35 |
| AGE | -0.19 | -0.22 | -0.20 | -0.45 | 0.29 | 0.06 | -0.30 | 1.00 | 0.37 |
| SEX | -0.60 | 0.03 | -0.19 | -0.18 | -0.07 | -0.20 | -0.35 | 0.37 | 1.00 |
|  |  |  |  |  |  |  |  |  |  |
| **2) change in positive interpretation bias from pre- to post training** | | | | | | |  |  |  |
|  | IMAGE | GENERATE | PRESENT | FEEDBACK | INTEN.RATIO | FREQ | STATUS | AGE | SEX |
| IMAGE | 1.00 | 0.27 | -0.03 | 0.25 | 0.04 | 0.23 | 0.40 | 0.29 | 0.12 |
| GENERATE | 0.27 | 1.00 | -0.49 | 0.49 | -0.36 | 0.42 | 0.44 | -0.32 | -0.16 |
| PRESENT | -0.03 | -0.49 | 1.00 | -0.78 | 0.50 | -0.41 | -0.47 | 0.41 | 0.54 |
| FEEDBACK | 0.25 | 0.49 | -0.78 | 1.00 | -0.50 | 0.36 | 0.41 | -0.34 | -0.47 |
| RATIO | 0.04 | -0.36 | 0.50 | -0.50 | 1.00 | -0.45 | -0.41 | 0.22 | 0.42 |
| FREQ | 0.23 | 0.42 | -0.41 | 0.36 | -0.45 | 1.00 | 0.73 | 0.04 | -0.39 |
| STATUS | 0.40 | 0.44 | -0.47 | 0.41 | -0.41 | 0.73 | 1.00 | 0.28 | -0.12 |
| AGE | 0.29 | -0.32 | 0.41 | -0.34 | 0.22 | 0.04 | 0.28 | 1.00 | 0.54 |
| SEX | 0.12 | -0.16 | 0.54 | -0.47 | 0.42 | -0.39 | -0.12 | 0.54 | 1.00 |
|  |  |  |  |  |  |  |  |  |  |
|  |  |  |  |  |  |  |  |  |  |
| **3) change in negative mood from pre- to post training** | | | | |  |  |  |  |  |
|  | IMAGE | GENERATE | PRESENT | FEEDBACK | INTEN.RATIO | FREQ | STATUS | AGE | SEX |
| IMAGE | 1.00 | 0.30 | -0.11 | 0.33 | -0.30 | 0.02 | 0.13 | -0.04 | 0.05 |
| GENERATE | 0.30 | 1.00 | -0.64 | 0.43 | -0.55 | -0.06 | 0.09 | -0.27 | 0.13 |
| PRESENT | -0.11 | -0.64 | 1.00 | -0.65 | 0.55 | -0.10 | -0.09 | 0.24 | 0.15 |
| FEEDBACK | 0.33 | 0.43 | -0.65 | 1.00 | -0.53 | 0.02 | 0.18 | -0.35 | -0.35 |
| RATIO | -0.30 | -0.55 | 0.55 | -0.53 | 1.00 | 0.02 | -0.07 | 0.29 | 0.21 |
| FREQ | 0.02 | -0.06 | -0.10 | 0.02 | 0.02 | 1.00 | 0.46 | 0.04 | -0.28 |
| STATUS | 0.13 | 0.09 | -0.09 | 0.18 | -0.07 | 0.46 | 1.00 | -0.11 | -0.19 |
| AGE | -0.04 | -0.27 | 0.24 | -0.35 | 0.29 | 0.04 | -0.11 | 1.00 | 0.38 |
| SEX | 0.05 | 0.13 | 0.15 | -0.35 | 0.21 | -0.28 | -0.19 | 0.38 | 1.00 |
|  |  |  |  |  |  |  |  |  |  |
| **4) difference in negative mood from pre- to post emotional challenge** | | | | | | |  |  |  |
|  | IMAGE | GENERATE | PRESENT | FEEDBACK | INTEN.RATIO | FREQ | STATUS | AGE | SEX |
| IMAGE | 1.00 | -0.04 | -0.09 | 0.35 | -0.35 | 0.15 | -0.09 | -0.66 | -0.10 |
| GENERATE | -0.04 | 1.00 | -0.75 | 0.23 | -0.59 | 0.20 | 0.45 | -0.20 | 0.18 |
| PRESENT | -0.09 | -0.75 | 1.00 | -0.43 | 0.48 | -0.15 | -0.33 | 0.29 | 0.01 |
| FEEDBACK | 0.35 | 0.23 | -0.43 | 1.00 | -0.50 | 0.13 | 0.30 | -0.50 | -0.54 |
| RATIO | -0.35 | -0.59 | 0.48 | -0.50 | 1.00 | -0.33 | -0.41 | 0.25 | 0.20 |
| FREQ | 0.15 | 0.20 | -0.15 | 0.13 | -0.33 | 1.00 | 0.34 | -0.18 | -0.27 |
| STATUS | -0.09 | 0.45 | -0.33 | 0.30 | -0.41 | 0.34 | 1.00 | 0.06 | -0.19 |
| AGE | -0.66 | -0.20 | 0.29 | -0.50 | 0.25 | -0.18 | 0.06 | 1.00 | 0.12 |
| SEX | -0.10 | 0.18 | 0.01 | -0.54 | 0.20 | -0.27 | -0.19 | 0.12 | 1.00 |
